# The burden, epidemiology, costs and treatment for Duchenne muscular dystrophy: an evidence review

**DOI:** 10.1186/s13023-017-0631-3

**Published:** 2017-04-26

**Authors:** S. Ryder, R. M. Leadley, N. Armstrong, M. Westwood, S. de Kock, T. Butt, M. Jain, J. Kleijnen

**Affiliations:** 1 Kleijnen Systematic Reviews Ltd., Unit 6, Escrick Business Park, Riccall Road, Escrick, York, YO19 6FD UK; 2 BioMarin Europe Ltd., 164 Shaftesbury Ave, London, WC2H 8HL UK; 3 School for Public Health and Primary Care, Maastricht University, Maastricht, The Netherlands

**Keywords:** Duchenne, DMD, Epidemiology, Prevalence, Incidence, Burden, Cost

## Abstract

**Background:**

Duchenne Muscular Dystrophy (DMD) is a rapidly progressive, lethal neuromuscular disorder, present from birth, which occurs almost exclusively in males. We have reviewed contemporary evidence of burden, epidemiology, illness costs and treatment patterns of DMD.

This systematic review adhered to published methods with information also sought from the web and contacting registries. Searches were carried out from 2005 to June 2015. The population of interest was individuals with clearly defined DMD or their carers.

**Results:**

Nine thousand eight hundred fifty titles were retrieved from searches. Fifty-eight studies were reviewed with three assessed as high, 33 as medium and 22 as low quality. We found two studies reporting birth and four reporting point prevalence, three reporting mortality, 41 reporting severity and/or progression, 18 reporting treatment patterns, 12 reporting quality of life, two reporting utility measures, three reporting costs of illness and three treatment guidelines.

Birth prevalence ranged from 15.9 to 19.5 per 100,000 live births. Point prevalence per 100,000 males was for France, USA, UK and Canada, 10.9, 1.9, 2.2 and 6.1 respectively. A study of adult DMD patients at a centre in France found median survival for those born between 1970 and 1994 was 40.95 years compared to 25.77 years for those born between 1955 and 1969. Loss of ambulation occurred at a median age of 12 and ventilation starts at about 20 years. There was international variation in use of corticosteroids, scoliosis surgery, ventilation and physiotherapy. The economic cost of DMD climbs dramatically with disease progression – rising as much as 5.7 fold from the early ambulatory phase to the non-ambulatory phase in Germany.

**Conclusions:**

This is the first systematic review of treatment, progression, severity and quality of life in DMD. It also provides the most recent description of the burden, epidemiology, illness costs and treatment patterns in DMD. There are evidence gaps, particularly in prevalence and mortality. People with DMD seem to be living longer, possibly due to corticosteroid use, cardiac medical management and ventilation. Future research should incorporate registry data to improve comparability across time and between countries and to investigate the quality of life impact as the condition progresses.

**Electronic supplementary material:**

The online version of this article (doi:10.1186/s13023-017-0631-3) contains supplementary material, which is available to authorized users.

## Background

Duchenne muscular dystrophy (DMD) is a severe, rapidly progressive neuromuscular disorder which belongs to a group of inherited conditions typically characterised by muscle weakening leading to increased disability. There are many different types of muscular dystrophy which vary considerably in severity, age of onset and life expectancy [1]. DMD is the most common and severe affecting 15.9 to 19.5 per 100,000 live births [2, 3] . DMD occurs as a result of mutations in the dystrophin gene which leads to an absence or deficiency of the protein dystrophin and continuous degeneration of muscle fibres. Although primarily an X-linked condition affecting males, some female carriers are symptomatic for the disorder but usually exhibit a milder phenotype.

Initial symptoms such as delayed walking, frequent falls and difficulty running and climbing stairs tend to be first noticeable between the ages of 1 and 3 years with muscles around the calf, pelvis and thigh often affected first and appearing noticeably bulkier than normal. Children with DMD typically need a wheelchair by the age of 8–14 years, as muscle weakening results in loss of ambulation. Once a patient becomes wheelchair bound, certain comorbid complications progress more rapidly including scoliosis and muscular contractures. Scoliosis, which causes the spine to curve sideways and/or forward or backward, leads to additional orthopaedic problems as one shoulder or hip becomes higher than the other leading to potential respiratory problems as the chest cavity reduces. DMD patients can develop symptoms of cardiomyopathy in the late teens, although the disease in this organ has likely started to develop earlier. Cardiomyopathy causes the heart’s chambers to enlarge and the walls to get thinner and in the late-teens or early 20s the condition is associated with breathing problems and once the heart and respiratory muscles are damaged the condition becomes life-threatening. Even with medical care, most people with DMD die from cardiac or respiratory failure before or during their 30s.

Age at diagnosis was not often reported but studies in Italy and Australia reported mean age as 4 years [4, 5]. DMD can be suspected when a male child shows abnormal muscle function and hypertrophy but tends to be confirmed following additional clinical presentation (including distribution of weakness) and a complete medical and family history. Elevated levels of serum creatine kinase support further diagnostic work-up for DMD. Historically, diagnosis was confirmed by genetic testing and/or muscle biopsy [6] although in practice, muscle biopsy is rarely undertaken. If clinicians are not fully aware of the manifestations of DMD then delays in diagnosis are likely.

There is no cure for DMD and current treatment options focus on alleviation of symptoms and management of complications. There is a recognized urgent need for a therapy that can alter the fundamental course of DMD and findings from this study of burden, epidemiology, costs and treatment should inform and support any future research. This review was originally designed to support a value proposition for a specific new treatment for DMD and the authors feel that publication of findings at this time will be of both interest and importance for any new intervention designed to manage the condition.

## Methods

This systematic review adhered to published methods including those recommended by the Cochrane Collaboration [7] and the Centre for Reviews and Dissemination [8] (York, UK), in order to reduce the risk of bias and error. Information was sought from a literature search, web based searches and through contacting registries and patient organisations.

### Research questions

The remit of our review was to identify, collate and describe contemporary evidence of epidemiology (prevalence and mortality), burden (severity and progression), illness costs (direct and indirect) and treatment patterns (pharmacological and other) of Duchenne muscular dystrophy. Current guidelines were also scrutinised for the latest treatment recommendations.

### Literature searches

Searches were carried out from 2005 to June 2015 in 10 databases to identify information on the epidemiology, prevalence and burden of DMD. Guideline searches were undertaken to identify management and treatment of DMD. A pragmatic internet search was also carried out to look for sources to support evidence gaps in prevalence of DMD. Additionally, email alerts and RSS feeds were set up to ensure the latest research was not missed. Further details of searching methods including example search strategies can be found in Additional file 1: Appendix 1.

The main Embase strategy was independently peer reviewed by a second Information Specialist, using the Canadian Agency for Drugs and Technologies in Health (CADTH) checklist [9].

### Methods of study selection

Titles and abstracts identified through electronic database and web searching were independently screened by two reviewers (drawn from a team of SR, RL, AH, MB, WJ) in order to determine whether they met the criteria for inclusion in the review. During this initial phase of the screening process any references which obviously did not meet the inclusion criteria were excluded. Full paper copies were obtained for all of the remaining references. These were then independently examined in detail by two reviewers (drawn from the team above working in pairs). All papers excluded at this second stage of the screening process were documented along with the reasons for exclusion. With respect to both screening stages, any discrepancies between reviewers were resolved through discussion or the intervention of a third reviewer (SR or NA).

### Inclusion criteria

Details are reported in Additional file 2: Appendix 2. In summary, aside from prevalence studies, where the general population (or subsets thereof) was of interest, we included all studies which described the population as DMD, even if details on diagnostic methods were missing. We excluded any studies which only reported on mixed populations (e.g. included Becker Muscular Dystrophy (BMD) or other forms of non-Duchenne Muscular Dystrophy).

Epidemiology and burden of disease outcomes of interest were: point prevalence, birth prevalence, demographic characteristics, clinical characteristics of the disease, mortality, incidence/prevalence of comorbidities and progression of the disease.

Quality of life (QoL) outcomes of interest were: the impact of the disease on quality of life (of patient and caregiver) as measured using a generic and disease specific or symptom specific measures.

Cost of illness outcomes of interest included patient and caregiver costs.

We also sought information about current treatment guidelines and treatment patterns.

Case studies were only included where evidence gaps could remain after consideration of other study types. Countries of interest included those in European Union (EU), South America, North America, Japan and Turkey (following advice from content experts at BioMarin Pharmaceuticals). For guidelines, countries of interest were restricted to EU countries and North America.

The years of interest were 2005 to 2015 inclusive. Due to the large number of papers retrieved and in order to concentrate on the most recent evidence, we decided to focus on records from 2010 onwards. Where evidence gaps existed, we sought records from earlier dates…

Studies were not limited by language or publication status (unpublished or published).

### Methods of data extraction

Data extraction was performed by two reviewers independently (drawn from a team of SR, RL, AH, MB, WJ). Any discrepancies were resolved through discussion or through the intervention of a third reviewer (SR or NA). Exemplar data extraction sheets are presented in Additional file 3: Appendix 3.

### Quality of study reporting

Two reviewers (drawn from a team of SR, RL, AH, MB, WJ) independently assessed each of the studies using a recommended tool, STROBE [10]. Any discrepancies were resolved through discussion or the intervention of a third reviewer (SR or NA).

Results are presented in Additional file 4: Appendix 4.

## Results

**Fig. 1:**
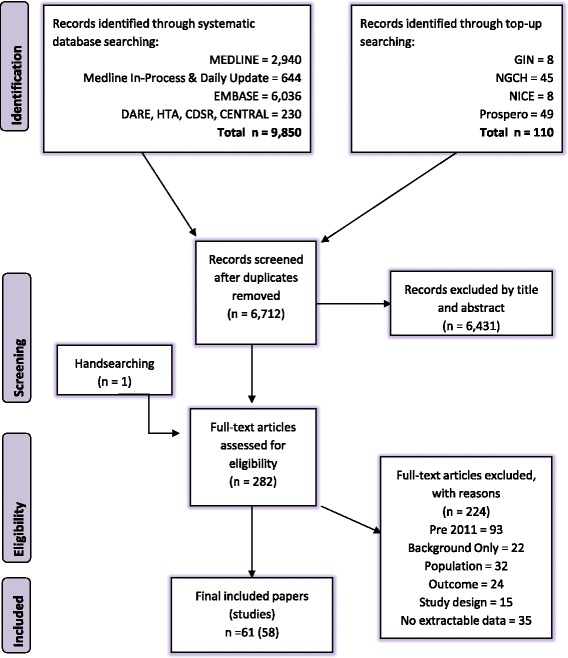
Flow diagram of included studies

In total, 9,850 titles were retrieved from the database searches and 110 titles were retrieved from the guidelines searches. After deduplication a total of 6,712 titles and abstracts were screened for relevance. Figure 1 summarises the flow of studies through the search and screening process. We excluded 6,431 articles during the title and abstract screening stage and 282 full papers of potentially relevant studies were selected for further examination (after having identified one additional guidelines paper [11] as a result of hand searching).

Of the 282 full papers that were screened, 221 did not meet the inclusion criteria and were excluded. Additionally, four papers with extractable data were part of the same study, meaning that three papers were treated as subsidiary papers to the main one.

Fifty-eight studies (from 61 papers) were therefore included in the review. These are summarised by research question in Additional file 5 with an indication of where follow-up data are available in longitudinal studies.

### Prevalence

Our review found a temporal trend from using both genetic testing and muscle biopsy towards only using genetic testing to identify cases of DMD. Population characteristics in earlier studies may therefore be different to those completed more recently.

Two studies reported birth prevalence and five studies reported point prevalence (see Additional file 4: Appendix 4 Table A8 for characteristics and Table 1 for results). Quality of study reporting was assessed in Additional file 4: Appendix 4 (Table A1 and Table A2). Both birth prevalence studies were judged to be of medium quality but lacked adequate description of study participants [2, 3]. Two of the point prevalence studies were judged to be of medium quality but again lacked an adequate description of study participants [12, 13]. The remaining three studies were judged to be of low quality [14–16]. Romitti [14] did not report an adequate description of the study design, nor did it fully describe the eligibility criteria or study participants. Mah [15] failed to adequately describe the eligibility criteria, outcomes or study participants. Bladen [16] also failed to provide adequate descriptions of eligibility criteria and study participants. Thus poor reporting makes it very difficult to assess possible changes in the way DMD has been defined over time.

**Table 1:** Summary of prevalence results by age grouping

Age Group	First author & publication year	Geography	Number DMD identified	Prevalence date	Mean prevalence per 100,000	Type of prevalence
Newborn	Mendell, 2012 [2]	Ohio	6	2007–2011	15.9	Birth
	Moat, 2013 [3]	Wales	72	1990–2011	19.5	
Boys aged 5 to 9	Romitti, 2015 [14]	USA	111	1991–1995	14.3	5 - year
			117	1996–2000	14.6	
			127	2001–2005	16.0	
			141	2006–2010	11.8	
Boys aged 5 to 24	Romitti, 2015 [14]	USA	389	2010	10.2	Point
Males under 18	Rasmussen, 2012 [13]	SE Norway	33	2005	16.2	
Males 0 to 24	Mah, 2011 [15]	Canada	529	2000–2009	10.3	
All Males	Norwood FL, 2009 [12]	Northern England	124	2007	8.3	
	Bladen 2013 [16]*	France	3337	2012	10.9	
		USA	2833	2012	1.8	
		UK	666	2012	2.2	
		Canada	1020	2012	6.1	

*Reported in Bladen [16] but based on web-based registry data

Of the studies reporting birth prevalence, one USA study by Mendell [2] carried out a study on newborn screening for DMD in one of the four main birthing hospitals in Ohio. Creatine kinase (CK) levels in newborn screening blood spots were measured followed by genetic analysis. The authors suggest this approach minimises false-positive testing. Birth prevalence was reported as 15.9 per 100,000 newborn males. A second study, Moat [3] reported on a newborn blood spot screening programme for DMD over a 21 year period in Wales, UK . Again, CK levels in newborn screening blood spots were measured followed by genetic analysis/muscle biopsy and adjusted for false negatives and cases identified where parents declined to participate in screening. Birth prevalence was reported as 19.5 per 100,000 new-born males.

Of the studies reporting point prevalence, a study by Bladen [16] reported on TREAT-NMD, a worldwide network for neuromuscular diseases which supports new therapies for patients . The network was reported to have many functions including clinical and epidemiological research. From the figure presented, the number of patients per country in the national DMD registry can be estimated and the point prevalence calculated. For France, USA, UK and Canada the point prevalence of DMD was calculated as 10.9, 1.9, 2.2 and 6.1 per 100,000 males, respectively. Mah [15] reported on a population based study of dystrophin mutations in Canada. Of the 773 individuals with dystrophinopathy as confirmed by genetic testing (97%), muscle biopsy (2%), or family history (1%), 529 had DMD. Point prevalence of DMD was reported as 10.3 per 100,000 males aged 0–24 in Canada based on the 2006 consensus. Rasmussen [13] reported on children with neuromuscular disorders from a region of South Eastern Norway. Diagnosis was confirmed by genetic testing and/or muscle biopsy. The point prevalence of DMD was 16.2 per 100,000 males under 18 years of age in this region reported on 1^st^ July 2005. Romitti [14] presented population based prevalence estimates for DMD and BMD in 6 US states based on the Muscular Dystrophy Surveillance, Tracking, and Research Network (MD STARnet) as established by the Centers for Disease Control and Prevention. Diagnosis of DMD was based on symptoms and age at onset, creatine kinase value, results of dystrophin mutation analysis testing, muscle biopsy reports, and family history. Point prevalence of DMD was 10.2 per 100,000 males aged 5–24 in 2010.

Few studies reported the prevalence of DMD in relation to an all age male population. However, we did find one study by Norwood [12] which reported a detailed population study of patients with genetic muscle disease in northern England. Although outside of our inclusion criteria, it presented most recent data on total population (without age restriction). The point prevalence was reported as 8.3 per 100,000 males on 1^st^ August 2007 based on 124 cases identified through genetic testing and muscle biopsy.

### Mortality

We identified three studies reporting information on the survival of DMD patients [17–19]. Quality of study reporting was assessed in Additional file 4: Appendix 4 (Table A3). Both Rall [19] and Kieny [20] were found to be of medium quality but neither provided an adequate description of study participants. Passamano [18] was assessed as low quality as it failed to adequately describe study design, outcomes or study participants and it was unclear if the study population was representative of the target population.

These three European long term retrospective cohort studies have each traced patients over a minimum of 30 years. All three studies (one each from Italy, France and Germany) reported median survival between 24 and 26 years. In the French study by Kieny [17, 20], median survival (calculated using the Kaplan-Meir model) was reported as 25.8 years for patients born between 1955 and 1969 and 40.9 years for patients born after 1970; the authors suggested that this difference was linked to greater availability of ventilator assistance through tracheotomy in the later birth cohort. In an Italian study by Passamano [18], the percentage overall mortality was assessed for patients when aged 20 and 25 born in either the 1960s, 1970s or 1980s. The study found that for those born in the 1960s, 76.7% would have died by the age of 20 and 86.5% by the age of 25; for those born in the 1970s, 46% would have died by the age of 20 and 69.4% by the age of 25; for those born in the 1980s, 40.2% would have died by the age of 20 and 50.8% by the age of 25. A study in Germany by Rall [19] also looked at patients born in the 1970s and found that median survival was 24 years, although this finding was sensitive to diagnosis method in that subjects with only a clinical diagnosis (as opposed to molecular testing) had a higher (67%) chance of reaching 24 years. Details of studies reporting mortality are set out in Additional file 4: Appendix 4 Table A9 with results in Table 2.

**Table 2:** Mortality results

First author & publication year	Name of subgroup	Country	Number of cases	Median survival	Number of deaths	% overall mortality
Kieny, 2013 [17]	Born 1955–1994	France	119	NR	55	27.6
	Born 1955–1969		43	25.77 years	NR	NR
	Born 1970–1994		76	40.95 years	NR	NR
Passamano, 2012 [18]	Born 1961–1990	Italy	516	NR	NR	NR
	Born 1961–1970 (age 25)		NR	NR	NR	86.5
	Born 1971–1980 (age 25)		NR	NR	NR	69.4
	Born 1981–1990 (age 25)		NR	NR	NR	50.8
	Born 1961–1970 (age 20)		NR	NR	NR	76.7
	Born 1971–1980 (age 20)		NR	NR	NR	46
	Born 1981–1990 (age 20)		NR	NR	NR	40.2
Rall, 2012 [19]	Born 1970–1980	Germany	67	Median survival was 24 years (21.3–26.7 CI) for patients diagnosed with molecular testing (*n* = 67). The probability of reaching 24 years was 67% for subjects with DMD diagnosed clinically only.	NR	NR

### Severity and progression

Forty-seven studies reported some information relating to the severity of DMD and/or its progression and 27 of these reported that they identified cases using genetic testing. The quality of reporting of these studies was recorded in Table A4. Three studies were judged to be of high quality [21–23] satisfying all the criteria. The remainder of the studies were medium and low quality, typically not providing adequate information on study participants leading to uncertainty as to whether the population was representative of the target population.

Study characteristics are set out in Additional file 4: Appendix 4 Table A10. Considerable variation in the methods used to measure severity was identified. We found considerable heterogeneity between studies in terms of criteria used to assess ambulatory status, wheelchair usage, mobility, scoliosis, cardiac and respiratory function and intelligence. Two studies reported the distribution of general severity i.e. a summary measure of disease status in the DMD population, both of which were based on ambulatory status as described by Bushby et al [6]. There was a far higher percentage of the German population [23] in the most severe equivalent category i.e. late non-ambulatory and non ambulatory with confinement (stages 4 and 5): 47.6% versus 35.8% in the US [24]. It is not clear why this should be the case, although it should be noted that the German study was of better quality and was published more recently. Results are set out in Additional file 4: Appendix 4 Table A11.

Ten studies reported cross-sectional data on loss of ambulation, either as the percentage who have already lost ambulation or mean age at loss of ambulation. Percentage loss of ambulation varied from 32.6% in a study of the whole DMD population across four continents (Bello [25]) to 56.4% in a Japanese study (Nakamura [26]) although age of participants was reported in neither study. The relationship with age was shown clearly in a US study by Mayer [27] in that there was no loss of ambulation before age 8 years and progressive loss until age 16–18 years, after which loss was 100%. One French study (Martigne [28]) reported a mean age of ambulation loss of 10 years. Unsurprisingly similar results were found in six studies reporting wheelchair use, four as percentage [29–32] and two as time to first use [19, 33]. Percentage use and time to use were unsurprisingly similar to those for loss of ambulation.

Eight studies reported mean six minute walking distance (6MWD plus, in some cases, other measures of mobility. 6MWD varied from 288.7 m reported in Pane [34] for those able to walk less than 350 m and aged at least 7 years to 428.7 m in those able to walk at least 350 m in the same age group. The only international study had an estimate of 361.1 m for those at least 5 years old (McDonald [22]).

The mean time to climb four stairs was varied between 2.5 s for those less than 7 years old to 6.6 s for those at least 7 years old in the international study (McDonald [22]). In the same study, mean 10 m run/walk time was 4.8 and 7.1 s respectively.

The percentage of the DMD population with scoliosis was reported in four studies from three countries and one multinational study. The percentage of the DMD population with scoliosis varied between 3.9% in a Japanese study of males with no age restrictions (Nakamura [26]) and 52.1% in a French study of boys ranging between 6 and 19 years old (Khirani [35]). The variation with stage of disease was shown in a multinational study in that the percentage was lowest at 16.6% in those in the early ambulatory stage (median age 7.2 years) and highest at 77.6% in the late non-ambulatory stage (median age 19.9) (Janssen [36]).

Cardiac function or percentage with cardiomyopathy was reported in six studies. Mean shortening fraction varied from 21.2% in the whole DMD population (left ventricular shortening fraction) from the US study by Ashwath [37] to 35% for those at least 10 years old from a US study by Thomas [38]. This variation appears to be consistent with age and thus disease stage. Percentage with cardiomyopathy varied similarly from 21% in younger boys (mean age (sd); 7.2 (2) years) (Thomas [38]) to 57.3% in a DMD population aged 10 years or more (Ashwath [37]). One study showed inter-country variation from 41.9% in Denmark to 52.4% in the UK in adult populations (Rodger [39]). It is not known whether demographic differences between populations may explain this difference.

Respiratory function, whether measured by percentage on assisted ventilation, time to introduction of ventilation, percentage of predicted Forced Expiratory Volume (ppFEV1) or percentage of predicted Forced Vital Capacity (ppFVC), was reported in 14 studies. The percentage of all DMD patients on assisted ventilation varied very widely from 0% in a Brazilian study of boys (mean age 11 years) by de Moura [29],0.7% in a multinational European study (mean age 13 years) by Vry [40] and 22% in a Japanese study (mean age not reported but most individuals described as less than 20 years old) (Nakamura [26]). Variation by disease progression was shown in the US study by Mayer [27] with a gradual decline from 126.6% predicted forced vital capacity (FVC) in the under 6 years age group to 7.3% predicted FVC in those aged 20 to 22 years.

Only one study (*n* = 4) by Khirani [35] in France reported the annual change in percent predicted Forced Vital Capacity (ppFVC). They found a 4.9% decline in respiratory function for patients with a mean age at baseline of 11.6 years. The percentage on assisted ventilation and age to start of ventilation after long term follow-up were reported in three moderate sized studies (no distinctions were made between night-time, daytime or continuous ventilation). After a mean follow-up of 18.3 years Martigne [28] found that 20% of study participants in France (mean age at baseline 13.0 years) were on assisted ventilation and the mean age for start of assisted ventilation was 16.8 years. In another French study, Kieny [17] claimed a follow up of 30 years, although the mean follow-up duration was not reported, and reported a much higher percentage of participants on assisted ventilation (65%) with a median age at start assisted ventilation of 20.1 years. The percentage on ventilation was also found to have increased from 60% before 1970 to 83% during and after 1970 with a decrease in the age at start of assisted ventilation from 20.1 to 18.3 years. The median age at start of assisted ventilation was essentially the same (i.e. 20 years) as that reported in the German study by Rall [19]. The authors of this study suggested that prolonged survival of DMD patients born after 1970 was directly associated with increased use of ventilation with tracheotomy especially when performed early. This was the only study to have made such a claim.

Two studies reported measures of intelligence, one reporting a mean score of 86.4 (compared to a mean score of 107.7 for a non-DMD group) on the Wechsler Intelligence Scale for Children-Revised in boys between 6 and 12 years (Lorusso [31]) and one reporting a mean score of 89.5 (compared to 100 for a non-DMD group) on the Bayley III cognitive composite instrument is in boys younger than 3 years from the US (Connolly [41]).

Progression of DMD can be measured in various ways. Those that are reported in the literature include changes in ambulatory status, ambulatory capacity (including 6MWD), and respiratory function.

Five studies followed up ambulatory status for those with DMD. Three of these studies began with those that were ambulant, two that followed up for 3 years in Italy by Pane [34] and in Italy and Belgium by Pane [42] and one that followed up for 7 years in the UK by Ricotti [43]. A study by Mah [44] followed up ambulant and non-ambulant boys for 1 year; this was an international study where information was not reported by country. Finally, Soderpalm [45] followed up anyone with DMD regardless of ambulatory status for 4 years in western Sweden.

In a study of boys in Italy (mean age 8.2 years), Mazzone [46] found a 3% loss in ambulation at 1 year follow-up. Over 3 years, the percentage who lost ambulation varied from 5.2% for those who with baseline 6MWD of ≥ 350 m and ≤ 7 years old to 64% with baseline 6MWD of <350 m and were ≥ 7 years old, reported in the follow up study by Pane [34] . In this study, loss of ambulation after 3 years, for combined sub groups, was 29%. In a study of DMD in Italy and Belgium, loss of ambulation was reported as 2.1% after 1 year in those who could originally walk ≥100 m (mean age at baseline 7.9 years) (Pane [42]) Mah [44] reported that, after 1 year, the percentage loss of ambulation increased from 43% at baseline to 57.1% at follow-up (mean age 12.0 at baseline). In a Swedish study, Soderpalm [45] for 18 patients aged between 2 and 19 years reported proportions of non-ambulant increasing from 22 to 50% over a 4-year mean follow up period. Therefore, it appears that participants in the Mah study may have been at an earlier stage in the disease. Median age for loss of ambulation was estimated at between 12 and 14 years by Ricotti [47] in the UK. Results are set out in Table 3 with changes in 6MWD in Table 4.

**Table 3:** Change in loss of ambulation

Country/countries	Name of subgroup	First author & publication year	Sample size	Mean age	Loss of ambulation (%)	Time point for follow up	Loss of Ambulation (n)@FU	Loss of ambulation (%)@FU
Italy	Ambulatory <350 m(6MWT), <7y	Pane, 2014 [34]	9	5.8	0	3y	2	22.22
	Ambulatory ≥350 m(6MWT), <7y		19	6.16			1	5.26
	Ambulatory <350 m(6MWT), ≥7y		25	9.87			16	64.00
	Ambulatory ≥350 m(6MWT), ≥7y		43	8.9			5	11.63
	Ambulatory-boys		96	NR		1y	3	3.00
						2y	16	17.00
						3y	27	29.00
Italy; Belgium	Ambulatory ≥100 m	Pane, 2014 [42]	191	7.9		1y	4	2.10
NR	Boys	Mah, 2012 [44]	340	12.0	43		194	57.06
Sweden	All DMD	Soderpalm, 2012 [45]	24	NR	17	4y	9	38.00
UK	Boys	Ricotti, 2012 [47]	400		NR	7Y	NR	Median loss of ambulation was 14 years in Daily versus 12 years in Intermittent prednisolone

**Table 4:** Change in 6MWD

Country/countries	Name of subgroup	First author & publication year	Sample size	Mean age	Mean 6MWD metres	6MWD (sd)	Time point for follow up	Mean 6MWD metres @FU	6MWD metres @FU (sd)
Australia; Belgium; Canada; France; Germany; Italy; Israel; Spain; Sweden; UK; USA	Ambulatory > =5y	McDonald, 2013 [22]	57	8.3	361.1	87.5	1y	317.4	152.3
	Ambulatory-Steroid treated, <7y		6	NR	382.8	42.7		416.9	35.1
	Ambulatory-Steroid treated, > = 7y		34		356.1	93.6		297.1	154.5
	Ambulatory-Steroid-naïve, <7y		8		366.3	63.5		341.4	163.9
Italy	Ambulatory < =7y, Continuous steroids	Mazzone,2014 [74]	NR		NR	NR		18.8	48.2
	Ambulatory <350 m(6MWT), <7y	Pane, 2014 [34]	9	5.8	307.9		3y	−49.3	173
	Ambulatory ≥350 m(6MWT), <7y		19	6.16	407.2			19.1	76.8
	Ambulatory <350 m(6MWT), ≥7y		25	9.87	288.7			−199.2	121.8
	Ambulatory ≥350 m(6MWT), ≥7y		43	8.9	428.7			−115	136.5
	Ambulatory-boys		96	NR	NR		1y	−15.8	77.3
							2y	−58.9	125.7
							3y	−104.22	146.2
		Mazzone,2014 [74]	106	8.3			1y	−25.8	74.3
		Mazzone,2014 [46]	113	8.2				−22.7	81
							2y	−64.7	123.1
	Ambulatory boys-None or intermittent steroids	Mazzone,2014 [74]	51	NR			1y	−40.6	82.2
	Ambulatory boys-Continuous steroids		55					−12.1	64.1
	Ambulatory boys < =7y		35					−7.8	63.9
	Ambulatory boys >7y		71					−42.3	73.9
	Ambulatory boys < =7y, None or intermittent steroids		NR					−0.44	73.7
	Ambulatory boys >7y, None or intermittent steroids							−66.4	77.7
	Ambulatory boys >7y, Continuous steroids							−23.6	65.9
Italy; Belgium	Ambulatory ≥100 m	Pane, 2014 [42]	191	7.9	378.06	74.13		−10.19	69.33
	Ambulatory ≥100 m, <7y		80	5.84	383.09	64.4		27.37	53.02
	Ambulatory ≥100 m, >7y		111	9.38	374.43	80.5		−37.25	67.21
	Ambulatory ≥100 m-Duplications		15	8.6	420.26	85.3		4.37	54.14
	Ambulatory ≥100 m-Point mutations		44	7.5	393.62	70.53		−0.84	53.6
	Ambulatory ≥100 m-All deletions		132	7.96	368.07	71.93		−14.95	75.09
	Ambulatory ≥100 m-Deletions eligible for skipping exon 44		18	8.2	398.16	65.28		−11.78	54.89
	Ambulatory ≥100 m-Deletions eligible for skipping exon 45		15	8.4	334.46	72.46		−21.6	111.76
	Ambulatory ≥100 m-Deletions eligible for skipping exon 46		7	7.3	335.3	85.47		0.83	24.51
	Ambulatory ≥100 m-Deletions eligible for skipping exon 50		9	7.4	358.63	77.11		−7.56	44.84
	Ambulatory ≥100 m-Deletions eligible for skipping exon 51		27	7.7	362.66	62.26		−21.59	76.33
	Ambulatory ≥100 m-Deletions eligible for skipping exon 53		28	8.6	344.11	67.16		−34.18	77.99
US	Ambulatory-boys	Henricson, 2012 [75]	17	NR	352	87		−73	135
		Henricson, 2013 [21]	24	7.9	369.5	79.3		−53.67	25.96

We found no evidence on the impact of specific mutations on severity/disease progression.

### Treatment patterns

We found 18 studies reporting treatment patterns for DMD patients. One study was assessed as high quality [23], 12 were assessed as medium and five as low (see Additional file 4: Appendix 4 Table A5) [4, 35, 39, 40, 47]. Typically low and medium quality studies neglected to report a description of the study participants or outcomes.

This section reports on different treatment regimens reported in different studies. Ideally this would be linked to experiences in terms of clinical outcomes; however, this is not possible because of the considerable variation in reporting of outcomes as well as heterogeneity of populations considered. Fourteen studies reported levels of corticosteroid usage, with a further four studies reporting on different aspects of care. International variations in use of corticosteroids, scoliosis surgery, ventilation and physiotherapy were found.

**Fig. 2:**
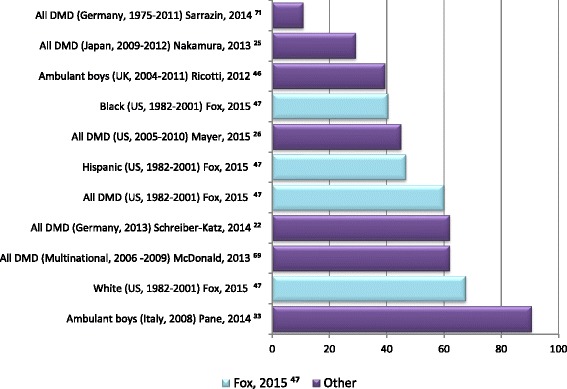
Variation in corticosteroid use

Usage of corticosteroids was found to vary by ethnicity with 67.6% of white American DMD patients having this treatment as opposed to only 40.5% of black American DMD patients (Fox, [48]). Characteristics of the studies providing evidence for corticosteroid use are set out in Additional file 4: Appendix 4 Table A12 with variation in use percentages shown in Fig. 2.

We found a number of studies reporting uptake of non-drug therapy. Uptake of scoliosis surgery was reported in one French study reporting that 52% of DMD patients underwent this surgery between 2001 and 2011 (Khirani [35]).

Another French study by Kieny [17] was the only one to report on uptake of ventilation. The prime focus of the study was to assess life expectancy over the period 1981 to 2011 and the study therefore relates to DMD at all ages. Kieny and others have suggested that ventilator assistance, mostly through tracheotomy, prolongs life expectancy. Although the numbers of cases were fairly low, only 27.9% of patients born prior to 1970 underwent tracheostomy, whereas this proportion had risen to 47.8% for patients born after 1980 (an even higher percentage was recorded for patients born between 1970 and 1980 at 58.5%).

Rodger [39] reported the most extensive results for non-pharmacological management of DMD patients. The study not only compared and contrasted treatment uptake in Germany, UK, Denmark and Eastern Europe but also did so separately for children and non-ambulatory adults. Use of physiotherapy services was particularly high in Germany and Denmark with 93.2 and 87.8% of non-ambulatory adults receiving this service, respectively. The comparative percentage for Eastern Europe was 51.3% with the UK only achieving 21.4%. A similar picture was evident for services received by children with DMD where utilisation was 91.8 and 93.3% in Germany and Denmark respectively but only 73.5 and 55% in Eastern Europe and UK respectively. These findings should also be considered alongside the reported weekly utilisation of these services, where the UK also has the lowest levels.

Rodger [39] also reported comparative experience of regular assessments/check-ups for non-ambulatory adults with DMD from UK, Denmark and Germany. The greatest variation was evident for 6 monthly lung function assessment with 45.2% seen in UK but only 7% in Denmark, 6 monthly cardiac function assessment with 33.8% uptake in Germany but only 9.5% in UK and hospital-based planned check-ups with 67.3% in Germany and 25.3% in Denmark. This points to considerable heterogeneity of care patterns from country to country.

### Quality of life/utility

Thirteen studies reported either HRQoL (see Additional file 4: Appendix 4 Table A13) or utilities (see Additional file 4: Appendix 4 Table A14). Parent proxy scores, where collated, were similar to directly elicited values. The quality of reporting of HRQoL studies is presented in Additional file 4: Appendix 4 Table A6. There are 2 high quality studies [21, 23], eight medium quality studies and three low quality studies [49–51]. Typically low and medium quality studies neglected to report a description of the study participants, outcomes or eligibility criteria.

The most frequently used tool for measuring HRQoL was the PedsQL, used in five studies. Three of these studies were conducted in the USA, the largest (*n* = 406) by Uzark [52] in four age groups. Bendixen [53] used two age groups (cut-off at 10 years) and Lim [50] for a cohort of boys and their parents (as proxies). Henricson [21] focused on those who were ambulatory, whereas Schreiber-Katz [23] covered the whole DMD population and subdivided by stage according to ambulatory ability. All studies provided at least the total score and two studies, by Schreiber-Katz [23] and Uzark [52] compared the DMD individual value with one elicited from the parent as proxy. Henricson [21] also used PODCI alongside PedsQL.

One study by Pangalila [54] in adults only, in the Netherlands, compared two different instruments, SF-36 and World Health Organization Quality of Life instrument (WHOQOL-BREF) as well as reporting the Fatigue Severity Score (0 to 5 scale) and Hospital Anxiety and Depression Scale (HADS) (0 to 21 scale). Simon [55] reported the Life Satisfaction Index for Adolescents (LSI-A) in four age groups of boys in Brazil, Baiardini [56] reported the Children Health Questionnaire - Parent Form 50 in Italian boys, Bendixen [51] reported the CAPE (0 to 5 scale) in the US and Canada, de Moura [29] reported the Autoquestionnaire Qualité de vie Enfant Imagé (AUQEI) in Brazil and Houwen-van Opstal [57] reported the KIDSCREEN-52 physical domain in the Netherlands. Interestingly, Pangalila [54] concluded that adults with DMD are ‘generally satisfied with their overall quality of life.’ Also, there was little variation according to stage as shown in both Houwen-van Opstal [57] on the KIDSCREEN instrument and Simon [55] on the LSI-A instrument with no clear trend.

Two papers reported utility values for DMD patients. One large multinational study Landfeldt [58] (*n* = 770) estimated Health Utilities Index (HUI) values (from both the boys and parents perspective) in four countries, Germany, Italy, the UK and the US. In this study the average for all DMD boys was 0.48 (considerably lower than perfect health (HUI = 1)) but inter-country variation was not large (from 0.43 in the UK to 0.52 in Italy). The other study, reporting utility values, Pentek [49], used EuroQol – 5 Dimensions – 5 Levels (EQ-5DL) for 57 boys with DMD in Hungary. However this study was judged of low quality reporting according to STROBE criteria, largely because of uncertainty surrounding representativeness of those evaluated.

### Costs of illness

We found one high quality study [23], one medium quality [58] and one low quality [32] study providing evidence of the cost of illness for DMD. The main features of these studies are set out in Additional file 4: Appendix 4 Table A15. Taken together, they represent a strong source of evidence of costs accrued at different stages of the condition and across different countries.

The high quality study assessed the burden of illness for German DMD patients and caregivers in 2013 [23]. This study provided an assessment of cost for customised severity groupings (based on Bushby et al [6]), thereby enabling better understanding of the costs associated with progression of the condition. For Schreiber-Katz [23], 2013 total direct medical costs ranged from 4,420 Euro (€) for (stage 1) patients to 68,968 Euro (€) for (stage 5) patients i.e. nearly a 16 fold increase. It is perhaps worth noting that the annual cost of hospitalisation represented between 7% (stage 4) and 14% (stage 1) of all direct costs in the Schreiber-Katz [23] study. Further subdivision of direct costs was provided including a detailed breakdown according to service headings (as opposed to staff group headings). The key variation in unit costs associated with progression relate to provision of medical aids costs for (stage 5) which were 104.5 times more than for (stage 1) and costs of respiratory management costs for (stage 5) which were 923.7 times more than for (stage 1). Results are set out in Table 5.

**Table 5:** Summary direct healthcare costs

Country	Name of subgroup	Cost Year	Currency	Cost of admission/hospitalisation		Cost of medication		All medical		All Direct Costs		First author & publication year
				Mean	LCI;UCI	Mean	LCI;UCI	Mean	LCI;UCI	Mean	LCI;UCI	
Germany	Stage 1 DMD Age 1 to 14	2013	Euro (€)	585	NR	172	NR	NR	NR	4220	NR	Schreiber-Katz, 2014 [23]
	Stage 2 DMD Age 3 to 14	2013		804	NR	373	NR	NR	NR	7629	NR	
	Stage 3 DMD Age 10 to 23	2013		NR	NR	344	NR	NR	NR	11666	NR	
	Stage 4 DMD Age 1 to 31	2013		1540	NR	319	NR	NR	NR	22989	NR	
	Stage 5 DMD Age 11 to 40	2013		6673	NR	550	NR	NR	NR	68968	NR	
	DMD Age 1 to 42	2013		1613	NR	330	NR	NR	NR	19346	NR	
	DMD Age 9 to 17	2012	US dollar ($)	2080	1020;4950	1020	770;2000	NR	NR	42360	38640;46880	Landfeldt(a), 2014 [58]
Italy	DMD Age 8 to 17	2012		1420	900;2470	1550	890;4650	NR	NR	23920	20420;28300	
UK	DMD Age 8 to 17	2012		2300	1500;3720	930	820;1070	NR	NR	54160	47310;63510	
US	DMD Age 9 to 17	2012		2220	2220;5050	2070	1720;2710	NR	NR	54270	48740;62220	
	DMD Age 0 to 64	2010		10012	NR	2144	NR	24007	NR	NR	NR	Larkindale, 2014 [32]

The study by Landfeldt [58] provided comparative direct costs for Germany, UK, US and Italy in terms of staff groupings as well as service headings. Most inter-country variation in large spend categories was found for physio/OT where spend in the US was 4.5 times that in Italy, psychology where spend in the US was 14.4 times that in either Italy or Germany, specialist physicians where spend in the US was 21.9 times that in Italy and visits to healthcare professionals where spend in the US was seven times that in Italy. The study by Larkindale [32] reported on medical costs but without comparative data or comparable cost categorisation.

Indirect costs were also quantified in each of the three cost of illness studies and expressed in terms of non-service costs and co-payments (see Table 6). Schreiber-Katz [23] assessed the costs of time off work and the impact on parents for each of the DMD stages in their study. They observed that (stage 5) patients had costs which were 2.5 times higher than (stage 2) patients in terms of costs of time off work and that (stage 1) patients had costs which were three times higher than (stage 5) patients in terms of impact on parents. Interestingly, taken together, these two forms of indirect costs represent a much greater cost than direct costs for (stage 1) patients (13,078 Euro (€) as opposed to 4,220 Euro (€)) and also a greater cost for (stage 5) patients (32,907 Euro (€) as opposed to 22,989 Euro (€)). The Landfeldt [58] study provided inter-country comparisons of indirect costs. There was broad similarity in terms of time off work and income loss. However, US funding mechanisms explain the relative high cost of insurance premiums. Loss of leisure time was costed as higher in Germany than in the other countries [58]. Information on co-payments was also provided as part of the Landfeldt [58] study. Italy has the highest co-payments of all four countries in each of the categories considered. No study estimated cost of lost productivity due to reduced life expectancy.

**Table 6:** Indirect costs of DMD; non service

Country: Subgroup: Cost year: Currency	Costs of time off work		Income loss		Insurance premiums		Loss of leisure time		Impact on parents		First author & publication year
	Mean	LCI;UCI	Mean	LCI;UCI	Mean	LCI;UCI	Mean	LCI;UCI	Mean	LCI;UCI	
Germany: DMD Age 9 to 17:2012:US dollar ($)	20770	17670;24250	1190	730;1880	150	60;290	17910	16210;20110	NR	NR	Landfeldt(a), 2014 [58]
Italy: DMD Age 8 to 17:2012:US dollar ($)	18220	15430;21380	620	310;1130	10	0;30	12440	10710;14980	NR	NR	
UK: DMD Age 8 to 17:2012:US dollar ($)	18700	16280;21150	750	440;1200	10	0;30	13590	12410;14980	NR	NR	
US: DMD Age 9 to 17:2012:US dollar ($)	21550	18490;24720	840	500;1360	6210	2820;14580	11700	10520;12630	NR	NR	
US:US DMD All Age:2010:US dollar ($)	NR	NR	15481	NR	NR	NR	NR	NR	NR	NR	Larkindale, 2014 [32]
Germany: DMD Age 1 to 42:2013:Euro (€)	21463	NR	NR	NR	NR	NR	NR	NR	7220	NR	Schreiber-Katz, 2014 [23]
Germany: Stage 1 DMD Age 1 to 14:2013:Euro (€)	NR	NR	NR	NR	NR	NR	NR	NR	13078	NR	
Germany: Stage 2 DMD Age 3 to 14:2013:Euro (€)	11100	NR	NR	NR	NR	NR	NR	NR	3855	NR	
Germany: Stage 3 DMD Age 10 to 23:2013:Euro (€)	NR	NR	NR	NR	NR	NR	NR	NR	8046	NR	
Germany: Stage 4 DMD Age 1 to 31:2013:Euro (€)	18734	NR	NR	NR	NR	NR	NR	NR	7044	NR	
Germany: Stage 5 DMD Age 11 to 40:2013:Euro (€)	28529	NR	NR	NR	NR	NR	NR	NR	4378	NR	

Social care costs were assessed in all three cost of illness studies for both services and equipment/adaptations – see Tables 7 and 8. The Schreiber-Katz [23] study provided evidence that informal carer and social care costs are positively associated with severity as measured by Stage of DMD. The costs of informal care time were 5.4 times higher for stage 5 patients than for stage 1. The costs of social care are also 5.4 times higher. In the international comparative study, Landfeldt [58] found broad similarities in the costs of informal carer time but the costs of home help; personal assistants etc. were notably higher in UK than in comparator countries. Information from Schreiber-Katz [23] on travel/car adaptations did not suggest a relationship with DMD progression and the comparative data provided by Landfeldt [58] suggested broad similarity in spend on equipment costs between UK, US and Germany with spend in Italy being noticeably lower.

**Table 7:** Social care expenses; services

Country: Subgroup: Cost year: Currency	Informal carer time/Care help		Home help, personal assistants, nannies, and transportation services.		Food, travel, diet etc.	Housing	Work/school assistance	Social care cost	First author & publication year
	Mean	LCI;UCI	Mean	LCI;UCI					
Germany: DMD Age 9 to 17:2012:US dollar ($)	18530	16440;20580	8920	6890;12400	NR	NR	NR	NR	Landfeldt(a), 2014 [58]
Italy: DMD Age 8 to 17:2013:US dollar ($)	13160	11270;15280	2740	1630;5380	NR	NR	NR	NR	
UK: DMD Age 8 to 17:2014:US dollar ($)	14340	13030;15990	19250	13240;28670	NR	NR	NR	NR	
US: DMD Age 9 to 17:2015:US dollar ($)	13370	12060;14930	7610	6210;10260	NR	NR	NR	NR	
US:US DMD All Age:2010:US dollar ($)	NR	NR	3189^a^	NR	6605	NR	NR	12939	Larkindale, 2014 [32]
Germany: DMD Age 1 to 42:2013:Euro (€)	21279	NR	NR	NR	NR	7102	883	30884	Schreiber-Katz, 2014 [23]
Germany: Stage 1 DMD Age 1 to 14:2014:Euro (€)	8303	NR	NR	NR	NR	2881	NR	11646	
Germany: Stage 2 DMD Age 3 to 14:2015:Euro (€)	8029	NR	NR	NR	NR	83	931	10684	
Germany: Stage 3 DMD Age 10 to 23:2016:Euro (€)	19532	NR	NR	NR	NR	3303	1980	29238	
Germany: Stage 4 DMD Age 1 to 31:2017:Euro (€)	31490	NR	NR	NR	NR	14359	1481	49834	
Germany: Stage 5 DMD Age 11 to 40:2018:Euro (€)	44443	NR	NR	NR	NR	17112	NR	62980	

^a^Described as care help

**Table 8:** Social care expenses; equipment and adaptations

Country: Subgroup: Cost year: Currency	Equipment cost		Moving or modifying home	Purchase or modifying motor vehicle	Travel/car adaptation	First author & publication year
	Mean	LCI;UCI				
Germany: DMD Age 9 to 17:2012:US dollar ($)	5560	4160;7460	NR	NR	NR	Landfeldt(a), 2014 [58]
Italy: DMD Age 8 to 17:2013:US dollar ($)	1850	970;4450	NR	NR	NR	
UK: DMD Age 8 to 17:2014:US dollar ($)	7520	5690;9790	NR	NR	NR	
US: DMD Age 9 to 17:2015:US dollar ($)	7930	6210;10260	NR	NR	NR	
US:US DMD All Age:2010:US dollar ($)	NR	NR	3050	1680	NR	Larkindale, 2014 [32]
Germany: DMD Age 1 to 42:2013:Euro (€)	NR	NR	NR	NR	1510	Schreiber-Katz, 2014 [23]
Germany: Stage 1 DMD Age 1 to 14:2014:Euro (€)	NR	NR	NR	NR	420	
Germany: Stage 2 DMD Age 3 to 14:2015:Euro (€)	NR	NR	NR	NR	1300	
Germany: Stage 3 DMD Age 10 to 23:2016:Euro (€)	NR	NR	NR	NR	4423	
Germany: Stage 4 DMD Age 1 to 31:2017:Euro (€)	NR	NR	NR	NR	2490	
Germany: Stage 5 DMD Age 11 to 40:2018:Euro (€)	NR	NR	NR	NR	915	

Finally, out-of-pocket expenses were considered in the Landfeldt [58] study as detailed in Table 9. These clearly represent a considerable cost of illness and there was a degree of similarity between countries.

**Table 9:** Out-of-pocket expenses

Country: Subgroup: Cost year: Currency	Patient/family cost	
	Mean	LCI;UCI
Germany: DMD Age 9 to 17:2012:US dollar ($)	4830	3150;7670
Italy: DMD Age 8 to 17:2012:US dollar ($)	4250	480;2350
UK: DMD Age 8 to 17:2012:US dollar ($)	3180	2020;5710
US: DMD Age 9 to 17:2012:US dollar ($)	5060	3130;8540

### Guidelines

Three key sources were identified in respect to guidelines for the treatment of DMD (see Table 10). In 2010, recommendations were made to consider glucocorticoids, including Deflazacort and Prednisone, as first line therapies for DMD patients of 2 years and over whose condition was not improving (Bushby [6]). Glucocorticoid therapy is highly recommended for patients of 6 years and over to slow the decline in muscle strength and function. It is also recommended that patients, in particular those with pre-existing risk factors, are monitored for side effects such as weight gain, growth retardation, bone demineralisation and increased fracture risk. Supplementary guidance for respiratory management of DMD patients was also published. Birnkrant [59] produced supplementary guidance for respiratory management of DMD patients which recommended equipment, procedures, tests, and diagnostic evaluations, emphasising the assessment of hypoventilation and the identification of specific thresholds for forced vital capacity (FVC), peak cough flow, and maximum expiratory pressure. More recently, results of an international collaboration were published (Kinnett [11]). These guidelines highlight the importance of a multidisciplinary approach to the care of DMD patients, addressing the primary and secondary manifestations of the condition including use of corticosteroids, coronary care, pulmonary care, physical therapy, surgical considerations and psychosocial care.

**Table 10:** Current treatment guidelines for DMD

Ref	Guideline title	Treatment type	Further details of treatment type	Population type to which recommendation applies	Considerations	Summary of recommendations	Other Guideline comments
Bushby(d), 2010 [6]	Diagnosis and management of Duchenne muscular dystrophy, part 1: diagnosis, and pharmacological and psychosocial management	Deflazacort	Start on 0 · 9 mg/kg/day	Age > =2, plateau or decline	Consider as first line when pre-existing weight and/or behavioural issues	Highly recommended for age > =6 and decline in age 2–6	Consider age, function (improving, plateau, declining), pre-existing risk factors, physician relationship with family. Ensure immunisation schedule is complete before initiating GCs. Alternative dosing strategies are also provided.
		Prednisone	Start on 0 · 75 mg/kg/day		First line unless pre-existing weight and/or behavioural issues favour deflazacort	Highly recommended for age > =6 and decline in age 2–5	
Birnkrant, 2010 [59]	The Respiratory Management of Patients With Duchenne Muscular Dystrophy	Respiratory management	Diagnostic testing, prevention, treatment, management under surgery	All DMD	All DMD	Recommendations on necessary equipment, procedures, tests, and diagnostic evaluations. It also provides a structured approach to the assessment and management of the respiratory complications of DMD, emphasising the assessment of hypoventilation and the identification of specific thresholds of forced vital capacity (FVC), peak cough flow, and maximum expiratory pressure	NR
Kinnett, 2015 [11]	A Simplified Guide to Comprehensive Care for Muscular Dystrophy	Multi-faceted	Diagnosis, support networks, corticosteroids, cardiac, assessment, rehabilitation, respiratory management, mental health, self help	All DMD	All DMD	Treatment recommendations include early start of corticosteroids, discussion of benefits and side effects by age of 3, evaluation of efficacy, discussion of longer term rationale for corticosteroid treatment. Other health maintenance strategies are promoted.	NR

## Discussion

We conducted a systematic review of contemporary (from 2010) evidence of burden, epidemiology, illness costs, treatment patterns and guidelines for DMD. In total, 9,850 titles were retrieved from searches. Fifty-eight studies were reviewed for reporting quality with three assessed as high quality, 33 as medium quality and 22 low quality.

Two studies reported birth prevalence from newborn screening programmes and five studies reported point prevalence. There appears to be a trend, over time, from using both genetic testing and muscle biopsy towards only using genetic testing in diagnosis which means that caution is required when comparing studies. This problem is exacerbated by inadequate descriptions of eligibility criteria and participants.

We found three studies on mortality [17–19]. People seem to be living longer with the condition. This is attributed to the widespread prescribing of corticosteroids, improved access to ventilation and the publication of more thorough and specific guidelines of care. For example, a French study [17] found that median survival for those born between 1970 and 1994 was 40.95 years compared to a mean lifespan of 25.77 years for those born between 1955 and 1969. Diagnosis method was also shown to be related to survival with molecular testing associated with a higher mortality than clinical only [19]. This is could have a number of implications, one being that the improvement in survival in those patients with true DMD, at least according to molecular testing, will never be known. As Kieny [17] points out in France: ‘Certainty of diagnosis was impossible before 1987, and therefore many patients did not initially have a definitive diagnosis.’ (p.444) Uncertainty in diagnosis would of course affect the ability to estimate prevalence as well.

We found forty one studies reporting aspects of disease severity and/or its progression. The prevalent DMD population has considerable dependency in that between 22% [45] and 56% [26] are likely to have lost ambulation and between 27% [24] and 57% [37] have cardiomyopathy. Severity clearly increases with age with a median of around 12 years for loss of ambulation [5] and about 20 years for start of ventilation [17]. Natural history is further explained by consideration of sub groups. The study by Pane [34] found that, over 3 years, the percentage loss of ambulation in those who were originally ambulant varied from 5.2% for those who could originally walk at least 350 m (<7 years old) to 64% for those who could originally walk less than 350 m (≥7 years old). One study undertaken in France [35] provided evidence of changing respiratory function which might be used to inform assessment of function/quality of life as disease progresses. Comparison of studies is hindered by variation in method of diagnosis and most studies inadequately reported participant characteristics.

Treatment patterns were reported in fourteen studies, which showed international variation in use of corticosteroids, scoliosis surgery, ventilation and physiotherapy. We also noted considerable variation in access to corticosteroids between different ethnic groupings as described by Fox [48]. Again, studies often failed to adequately report participant characteristics.

Thirteen studies reported either HRQoL or utilities. The most frequently used tool for measuring HRQoL was the PedsQL which was used in five studies [21, 23, 50, 52, 53] and, for utilities, HUI was calculated for Germany,Italy, UK and US populations in Landfeldt [58]. These measures could be considered when designing future studies although researchers should be aware that some measures reportedly correlate better with disease progression than others. For example there is evidence to suggest that the generic PedsQL does not correlate well with progression of disease in DMD [60]. There may be a trade-off between sensitivity of measurement tool and compatibility with historical research. However, it is also interesting to speculate that lack of change in self-reported quality of life with stage that was observed in two studies is not related to insensitivity of instrument, but reflects the stability irrespective of deterioration in physical status [55, 57]. This might reflect adaptation, which is perhaps why parents might produce lower estimates as shown in Houwen-van Opstal [57].

Indirect costs (due to loss of productivity) appear higher than direct costs (of health or social care) for early stage patients and late stage patients but not necessarily for intermediate stages [23], which highlights the importance of staging to inform co-ordinated financial planning of health and social care. However, these findings are based on only one study.

The main strength of our approach was that it used established systematic review methods to consider a broad range of characteristics of disease impact. The main potential limitation of our approach was in its restriction to published and unpublished evidence from 2011 to 2015. This restriction was imposed because all things being equal, contemporary evidence is of much greater relevance than historic evidence. Also more recent studies should have a better diagnosis procedure, distinguish better between BMD and DMD and be more representative of the DMD population. Nevertheless, although recency is important, it might be argued that our search missed older studies and we identified very few studies of prevalence, incidence and mortality which have been published since 2010. However, a comparison to two systematic reviews of epidemiology from 2014 (Theadom [61] and Mah [62]) revealed virtually no studies in the 5 years prior to 2010 (only a household survey in Egypt 2005 and an abstract of a study of practitioner-referrals in Portugal in 2006). A recent systematic review of cost of illness evidence in rare diseases (Angelis [63]) also failed to identify any contemporary cost of illness studies for DMD, which gives us some confidence that most relevant studies have been identified in our review. We were unable to identify any systematic reviews of treatment, progression, severity or utility, to the best of our knowledge, our study is the first to cover these aspects in a systematic way.

Evidence gaps (particularly in regard to prevalence, life expectancy and treatment patterns) might eventually be filled with the emergence of registries. TREAT-NMD is a web-based community of researchers and those with special interests in neuromuscular diseases which acts as a portal for registries. The network was launched in January 2007 and their website contains contact details for 49 separate national registries across all continents (http://www.treat-nmd.eu/ [64]). As more “real-time” information is collated it may become increasingly common to undertake prevalence studies using registry data. Other important sources include ongoing natural history studies run by The Cooperative International Neuromuscular Research Group (CINRG).

## Recommendations

We suggest that, as well as natural history studies, patient registries should be considered as a future source of data to estimate prevalence, treatment patterns, effectiveness and to explore variation in severity, progression and mortality. Registries offer a number of key advantages over other forms of primary research in that they largely use a consistent set of criteria (potentially at an international level), they can be up-to-date and they offer potential for cross-matching of patient characteristics and other clinical indicators. Feasibility studies should address coverage levels (as not everyone in a location may be registered) and also data quality assurance issues (e.g. to avoid double-counting of patients and/or means of updating for new cases and deaths).

We also recommend that future studies, which purport to measure overall burden of the condition, fully account for DMD in all age groups and severity/stages of disease. There are very few prevalence studies and no comparability between them because they relate to different denominator populations (typically defined by different age groupings). In particular, there is a need to focus on prevalence in relation to the whole male population, thereby reflecting the changing age profile of those with the condition. Such studies offer the best potential to fully capture burden levels in an entire economy or location and ultimately to improve clinical awareness.

Whilst we found reasonable quality evidence about the cost of illness, only one study conducted a between country comparison [58]. Also, there is a need to relate this to severity/stage of disease, thereby enabling researchers to fully capture the cost consequences of treatment modifications that alter progression of the condition (including survival). We would recommend further research into the implications of quality of life for comorbid conditions like scoliosis in patients with DMD, alongside associated costs. Similarly, quality of life of carers remains under-researched.

One final recommendation, which pertains to all study types, is greater standardisation of reporting: many studies suffered from poor reporting of eligibility criteria or study participant characteristics.

## Conclusions

From a systematic review, fifty eight studies (published since 2010) were found that examined DMD in terms of epidemiology, cost, quality of life and guidelines. There are important evidence gaps, particularly in measuring prevalence and mortality, although people seem to be living longer with the condition, which may be partially as a result of more widespread prescribing of corticosteroids, improved access to ventilation and development and publication of more specific and thorough care guidelines. Increased longevity means that studies of prevalence based only on younger populations will become less representative of the disease burden of DMD. Evidence for wider populations as opposed to specific age/ambulatory status sub groupings should become increasingly more relevant, with studies in older populations with advanced progression currently under-represented.

Disease severity in the prevalent DMD population also appears to be high in that at any given time and any given country between 22 and 56% are likely to have lost ambulation and between 27 and 57% have cardiomyopathy. Severity clearly increases with age with a median of around 12 years for loss of ambulation and about 20 years to start ventilation.

Comparability of evidence on changing prevalence and mortality is hampered by changing case definitions with a trend from using both genetic testing and muscle biopsy towards only using genetic testing.

Indirect costs are a significant feature of this condition and should have a role in informing appropriate care packaging and co-ordinated financial planning of health and social care. Per capita cost burden increases with disease progression. The main recommendations, arising from this systematic review, are for the increased collection and use of registry data to increase comparability across time and between countries.

## Additional files

Additional file 1: Appendix 1. Search strategies. (DOC 43 kb)

Additional file 2: Appendix 2. Inclusion criteria. (DOC 24 kb)

Additional file 3: Appendix 3: Data extraction sheet. (DOC 335 kb)

Additional file 4: Appendix 4. Study reporting quality (STROBE) and characteristics. (DOC 1545 kb)

Additional file 5: Summary of included studies by research question [64–73]. (DOC 462 kb)

## Abbreviations

6mwd: Six Metres Walking Distance
BMD: Becker muscular dystrophy
CADTH: Canadian Agency for Drugs and Technologies in Health
CAPE: Children’s Assessment of Participation and Enjoyment
CDSR: Cochrane database of systematic reviews
CENTRAL: Cochrane Central Register of Controlled Trials
CK: Serum creatine-kinase (CK)
DARE: Database of abstracts of reviews of effects
DMD: Duchenne muscular dystrophy
EQ-5D: EuroQol – 5 Dimensions
EQ-5D-5 L: EuroQol – 5 Dimensions – 5 Levels
ER: Emergency room
EU: European Union
GIN: International Guidelines Network Library
HRQoL: Health related quality of life
HTA: Health technology assessment
NHS EED: NHS economic evaluation database
NHS: National health service
NM: Neuromuscular
PedsQL: Paediatric Quality of Life
PODCI: The Pediatric Outcomes Data Collection Instrument
ppFEV_1_: Percentage of predicted Forced Expiratory Volume
ppFVC: Percentage of predicted Forced Vital Capacity
QoL: Quality of life
sd: Standard Deviation
STROBE: Strengthening the Reporting of Observational Studies in Epidemiology
TREAT-NMD: Translational Research in Europe–Assessment and Treatment of Neuromuscular Diseases
UK: United Kingdom
USA: United States of America
