# Supplementary material for: The burden, epidemiology, costs and treatment for Duchenne muscular dystrophy: an evidence review
Source: Orphanet J Rare Dis. 2017 Apr 26;12:79. doi: 10.1186/s13023-017-0631-3 (PMC5405509; doi:10.1186/s13023-017-0631-3)
Supplement: Supplementary file 1 — Search strategies. (DOC 43 kb) [file 13023_2017_631_MOESM1_ESM.doc]

# Additional file 1: Appendix 1: Search Strategies

Searches were carried out from 2005 to June 2015 in the following databases: Medline; Medline In-Process & Other Non-Indexed Citations; Medline Daily Update; Embase; Cochrane Database of Systematic Reviews (CDSR); Cochrane Central Register of Controlled Trials (CENTRAL); Database of Abstracts of Reviews of Effects (DARE); Health Technology Assessment Database (HTA); NHS Economic Evaluation Database (NHS EED) and PROSPERO (International Prospective Register of Systematic Reviews) to identify relevant information on the epidemiology, prevalence and burden of DMD.

Searches to identify international guidelines and guidance on the management of DMD were performed in: International Guidelines Network Library (GIN); National Guidelines Clearinghouse; NICE Guidance (National Institute for Health and Care Excellence).

To pick up the latest reviews on DMD, an email alert was also set up in The Cochrane Library using the strategy designed for The Cochrane Library and RSS feeds were set up in the websites <https://www.york.ac.uk/crd/>, <https://www.euroscan.org/> and <http://www.nihr.ac.uk/>.

Web based searching to identify useful sources of information on prevalence were carried out on:

- Centre for Disease Control and Prevention (searched 9/10/15) [http://www.cdc.gov/ncbddd/musculardystrophy/](http://www.cdc.gov/ncbddd/musculardystrophy/features/key-findings-population-duchenne.html)
- Treat NMD (searched 9/10/15) <http://www.treat-nmd.eu/>
- Social and Economic Burden and Health-related Quality of Life in patients with rare diseases in Europe BURQOL RD (searched 9/10/15) <http://www.burqol-rd.com/>.

These latter two sources included details for further information and were contacted on 9/10/15.

All identified references were imported into Endnote X7 software for further assessment and handling. Individual records within the Endnote reference libraries were tagged with searching information, such as searcher, date searched, database host, database searched, strategy name and iteration, theme or search question.

**Embase (Ovid):2005-2015/6/30**

**Searched 1.7.15**

1 DMD.ti,ab,ot. (6526)

2 (duchenne$ adj3 (syndrome$ or morbus)).ti,ab,ot,hw. (69)

3 ((duchenne$ or becker$ or backer$ or pseudo hypertrophic or pseudohypertrophic) adj3 dystroph$).ti,ab,ot,hw. (14041)

4 (cardiomyopathy adj2 dilated adj2 (x-linked or 3b)).ti,ab,ot,hw. (75)

5 ("duchenne/becker" or "duchenne-becker" or "DMD/BMD" or "BMD/DMD").ti,ab. (631)

6 Duchenne muscular dystrophy/ (10882)

7 Becker muscular dystrophy/ (2177)

8 or/1-7 (15453)

9 animal/ (1673161)

10 animal experiment/ (1860359)

11 (rat or rats or mouse or mice or murine or rodent or rodents or hamster or hamsters or pig or pigs or porcine or rabbit or rabbits or animal or animals or dogs or dog or cats or cow or bovine or sheep or ovine or monkey or monkeys).ti,ab,ot,hw. (5963610)

12 or/9-11 (5963610)

13 exp human/ (16094390)

14 human experiment/ (338643)

15 or/13-14 (16095833)

16 12 not (12 and 15) (4723494)

17 8 not 16 (12864)

**18 limit 17 to yr="2005 -Current" (6036)**

**Medline (Ovid): 2005 – 2015/06/Week 3**

**Searched: 1.7.15**

1 Muscular Dystrophy, Duchenne/ (3585)

2 DMD.ti,ab,ot. (4571)

3 (duchenne$ adj3 (syndrome$ or morbus)).ti,ab,ot,hw. (50)

4 ((duchenne$ or becker$ or backer$ or pseudo hypertrophic or pseudohypertrophic) adj3 dystroph$).ti,ab,ot,hw. (9157)

5 ("duchenne/becker" or "duchenne-becker" or "DMD/BMD" or "BMD/DMD").ti,ab. (507)

6 (cardiomyopathy adj2 dilated adj2 (x-linked or 3b)).ti,ab,ot,hw. (66)

7 or/1-6 (10097)

8 exp animals/ not (exp animals/ and humans/) (4061621)

9 7 not 8 (8335)

**10 limit 9 to yr="2005 -Current" (2940)**

**Medline In-Process Citations (Ovid): up to 2015/06/29**

**Medline Daily Update (Ovid): up to 2015/06/29**

**Searched: 30.6.15**

1 Muscular Dystrophy, Duchenne/ (4)

2 DMD.ti,ab,ot. (487)

3 (duchenne$ adj3 (syndrome$ or morbus)).ti,ab,ot,hw. (2)

4 ((duchenne$ or becker$ or backer$ or pseudo hypertrophic or pseudohypertrophic) adj3 dystroph$).ti,ab,ot,hw. (515)

5 ("duchenne/becker" or "duchenne-becker" or "DMD/BMD" or "BMD/DMD").ti,ab. (22)

6 (cardiomyopathy adj2 dilated adj2 (x-linked or 3b)).ti,ab,ot,hw. (4)

7 or/1-6 (706)

8 exp animals/ not (exp animals/ and humans/) (2635)

9 7 not 8 (704)

10 limit 9 to yr="2005 -Current" (644)

**Cochrane Database of Systematic Reviews (CDSR) (Wiley): 2005-Issue6/June 2015**

**Database of Abstracts of Reviews of Effects (DARE) (Wiley): 2005-Issue2/April 2015**

**Cochrane Register of Controlled Trials (CENTRAL) (Wiley): 2005-Issue5/May 2015**

**Health Technology Assessment database (HTA) (Wiley): 2005-Issue2/April 2015**

**NHS Economic Evaluation Database (NHSEED) (Wiley): 2005-Issue2/April 2015**

**Searched: 30.6.15**

#1 MeSH descriptor: [Muscular Dystrophy, Duchenne] this term only 71

#2 DMD 215

#3 (duchenne* near/3 (syndrome* or morbus)) 1

#4 ((duchenne* or becker* or backer* or pseudo-hypertrophic or pseudohypertrophic) near/3 dystroph*) 302

#5 ("duchenne/becker" or "duchenne-becker" or "DMD/BMD" or "BMD/DMD") 21

#6 (cardiomyopathy near/2 dilated near/2 (x-linked or 3b)) 1

#7 #1 or #2 or #3 or #4 or #5 or #6 Publication Year from 2005 to 2015 231

CDSR = 32

DARE = 5

CENTRAL = 191

HTA = 2

NHS EED = 0

**GIN (Internet): Up to 2015/06/30**

[**http://www.g-i-n.net/gin**](http://www.g-i-n.net/gin)

Searched 30.6.15

| **Search terms** | **Records** |
| --- | --- |
| DMD | 2 |
| duchenne | 5 |
| BMD | 0 |
| becker | 1 |
| **Total** | **8** |
| **Total after dedupe** | **5** |

**National Guidelines Clearinghouse (Internet): up to 2015/06/30**

[**http://www.guideline.gov/**](http://www.guideline.gov/)

Searched 30.6.15

| **Search terms in title** | **Records** |
| --- | --- |
| DMD OR “Duchenne muscular dystrophy” OR BMD OR “Becker muscular dystrophy” | 45 |
| **Total** | **45** |

**NICE (Internet): up to 2015/06/30**

[**http://www.nice.org.uk/**](http://www.nice.org.uk/)

Searched 30.6.15

| **Search terms in title** | **Records** |
| --- | --- |
| DMD OR duchenne OR BMD OR becker | 8 |
| **Total** | **8** |

**PROSPERO (Internet): up to 2015/06/30**

[**http://www.crd.york.ac.uk/prospero/**](http://www.crd.york.ac.uk/prospero/)

Searched 30.6.15

| All fields | Records |
| --- | --- |
| DMD OR duchenne OR BMD OR becker | 49 |
| Total | 49 |
