# Supplementary material for: The burden, epidemiology, costs and treatment for Duchenne muscular dystrophy: an evidence review
Source: Orphanet J Rare Dis. 2017 Apr 26;12:79. doi: 10.1186/s13023-017-0631-3 (PMC5405509; doi:10.1186/s13023-017-0631-3)
Supplement: Supplementary file 2 — Inclusion criteria. (DOC 24 kb) [file 13023_2017_631_MOESM2_ESM.doc]

# Additional file 2: Appendix 2: INCLUSION CRITERIA

Population

Population related to individuals with clearly defined DMD excepting incidence and prevalence studies where the population is the general population. Subgroups of interest were males, ethnic groups, deletions amenable to exon 51-skipping technology, ambulatory patients less than 7yrs, non-ambulatory with upper body functionality, non-ambulatory with compromised respiratory function, exon skipping groups/genotypes (e.g. as described), and BMD if described as a sub group of DMD.

Intervention and comparators

• Any

Outcomes

• Epidemiology and burden of disease: Incidence, prevalence, point prevalence, birth prevalence, demographic characteristics (e.g. age, height, weight, ethnicity), clinical characteristics of the disease (e.g. severity, day/night ventilation use, upper limb assessment, muscle strength tests, time function tests), mortality, incidence/prevalence of comorbidities and development of the disease (e.g. change in health status/clinical characteristics over time).

• HRQoL: Impact of the disease on quality of life (of patient and caregiver) as measured using a generic and disease specific or symptom specific measures e.g. SF-36, Paediatric Quality of Life Inventory TM Neuromuscular Module (PedsQL NM), the Children’s Assessment of Participation and Enjoyment (CAPE), The Paediatric Outcomes Data Collection Instrument (PODCI), NeuroQoL or DMD specific measure and impact on quality of life as measured using a Utility instrument e.g. EQ-5D.

• Cost of illness: (including patient and caregiver costs) including average annual costs per person, cost of health care and social care, cost of the disease, rate of use of resources (e.g. hospitalisations, office visits, ER visits, medication, other) and average annual indirect cost per person and cost to the patient.

• Current treatment guidelines and treatment options: including key clinical guidelines in North America and EU, treatment pathways, the current treatment options, recommendations regarding the use of therapies and prevalence of each treatment (percentage of patients are currently taking/prescribed each therapy).

Study design

• Observational studies for epidemiological characteristics including incidence and prevalence

• HRQoL or preference elicitation studies

• Cost of illness studies

• Guidelines and guidance for treatments, disease management and treatment pathways

• Case studies were only included if they informed key gaps in the evidence.

Geographical location

• Countries of interest included those in EU, South America, Japan, Turkey and North America. For guidelines countries of interest included EU and North America.

Language

• No restriction on language.

Additional factors

• Any studies found through reference checking, which fall outside of the date limit used for literature searches but are otherwise relevant, have been included.
