# Supplementary material for: The burden, epidemiology, costs and treatment for Duchenne muscular dystrophy: an evidence review
Source: Orphanet J Rare Dis. 2017 Apr 26;12:79. doi: 10.1186/s13023-017-0631-3 (PMC5405509; doi:10.1186/s13023-017-0631-3)
Supplement: Supplementary file 3 — Data extraction sheet. (DOC 335 kb) [file 13023_2017_631_MOESM3_ESM.doc]

# Additional file 3: Appendix 3: DATA EXTRACTION SHEET

| **STUDY CHARACTERISTICS** | |
| --- | --- |
| Study details | Endnote ID |
|  | First author and publication year |
|  | *Population studied (select one)* |
|  | Population of interest (All age, males, age-defined, MD, Mixed) |
|  | Minimum age (if age defined) |
|  | Maximum age (if age defined) |
|  | Country/countries (list separated by ";") |
|  | *Region (if specified)* |
|  | *Publication Type (select one)* |
|  | Reported name for Duchenne muscular dystrophy |
|  | Data collection period (xx/yyyy to xx/yyyy) |
|  | *Study design (select one)* |
|  | Study duration (years) |
|  | Study aims |
|  | Study conclusions |
|  | Inclusion criteria (semi colon separated) |
|  | Exclusion criteria (semi colon separated) |
|  | *Data source (select one)* |
|  | Data source name (e.g. database/survey name) |
|  | N population size |
|  | n for DMD included in study |
|  | Number lost to follow-up |
|  | Reasons for lost to follow-up (c,s,v) |
|  | Other EPI Study comments |

| **PATIENT CHARACTERISTICS** | |
| --- | --- |
| Study details | Endnote ID |
|  | First author and publication year |
|  | Country/countries (list separated by ";") |
| Demographics | Number of males |
|  | % males |
|  | Mean age |
|  | Median age |
|  | Age range [x,y] |
|  | Mean weight (kg) |
|  | Median weight (kg) |
|  | Weight range [x,y] |
|  | Mean height (cm) |
|  | Median height (cm) |
|  | Height range [x,y] |
|  | List of ethnic groups with % (format - White (25%); Black (30%) etc. ) |
|  | Consanguinity (description) |
|  | No with consanguinity |
|  | Demographics comments (if required) |
| Diagnosis | Mean age at diagnosis |
|  | Median age at diagnosis |
|  | Age range at diagnosis [x,y] |
|  | Description of diagnostic method (if referenced put Author, date e.g. Emery, 1997) |
|  | DNA/genetic testing (select one) |
|  | Muscle biopsy (select one) |
|  | Description of method for muscle biopsy |
|  | Diagnosis comments (if required) |
| Genotype | Exon with deletion 51 |
|  | Exon with deletion 51 (%) |
|  | Nonsense mutation in dytrophin gene (n) |
|  | Nonsense mutation in dytrophin gene (%) |
|  | Stop codon UGA (n) |
|  | Stop codon UGA (%) |
|  | Stop codon UAG (n) |
|  | Stop codon UAG (%) |
|  | Stop codon UAA (n) |
|  | Stop codon UAA (%) |
|  | Genotype comments (if required) |
| Severity (at baseline) | Overall severity type - authors description |
|  | List of severity groups with % (format - Mild (50%); moderate (27%); etc.) |
|  | Loss of Ambulation (n) |
|  | Loss of ambulation (%) |
|  | Wheelchair user (n) |
|  | Wheelchair user (%) |
|  | Periambulatory (n) |
|  | Periambulatory (%) |
|  | Use of mechanical support i.e. braces (n) |
|  | Use of mechanical support i.e. braces (%) |
|  | Assisted ventilation (n) |
|  | Assisted ventilation (%) |
|  | Respiratory infections (n) |
|  | Respiratory infections (%) |
|  | Feeding tube needed (n) |
|  | Feeding tube needed (%) |
|  | Mean 6MWD (metres) |
|  | Mean climb 4 stairs (sec) |
|  | Mean descend 4 stairs (sec) |
|  | Mean 10m run/walk (sec) |
|  | Mean supine to stand (sec) |
|  | Mean falls/day |
|  | Name of Measure of arm movement |
|  | Mean value of Measure of arm movement |
|  | Pulmonary function (FEV-1) |
|  | Pulmonary function (FVC) |
|  | Name of Cardiac function measure |
|  | Mean value of cardiac function measure |
|  | Scoliosis (n) |
|  | Scoliosis (%) |
|  | COPD (n) |
|  | COPD (%) |
|  | Name of Intellection measure (IQ) |
|  | Mean of intellection measure (IQ) |
|  | Severity at baseline comments (if required) |
| Severity at follow-up i.e. development of disease | Absolute or change from baseline (prioritised in that order) |
|  | Time point for follow up |
|  | List of severity groups with % (format - Mild (50%); moderate (27%); etc.) @ FU |
|  | Loss of Ambulation (n) @ FU |
|  | Loss of ambulation (%) @ FU |
|  | Wheelchair user (n) @ FU |
|  | Wheelchair user (%) @ FU |
|  | Periambulatory (n) @FU |
|  | Periambulatory (%)@FU |
|  | Use of mechanical support i.e. braces (n) @ FU |
|  | Use of mechanical support i.e. braces (%) @ FU |
|  | Assisted ventilation (n) @ FU |
|  | Assisted ventilation (%) @ FU |
|  | Respiratory infections (n) @ FU |
|  | Respiratory infections (%) @ FU |
|  | Feeding tube needed (n) @ FU |
|  | Feeding tube needed (%) @ FU |
|  | Mean 6MWD (metres) @ FU |
|  | Mean climb 4 stairs (sec) @ FU |
|  | Mean descend 4 stairs (sec) @ FU |
|  | Mean 10m run/walk (sec) @ FU |
|  | Mean supine to stand (sec) @ FU |
|  | Mean falls/day @ FU |
|  | Measure of arm movement @ FU |
|  | Pulmonary function (FEV-1) @ FU |
|  | Pulmonary function (FVC) @ FU |
|  | Cardiac function @ FU |
|  | Scoliosis (n) @FU |
|  | Scoliosis (%) @FU |
|  | COPD (n) @FU |
|  | COPD (%) @FU |
|  | Intellection measure (IQ) @ FU |
|  | Severity at follow up comments (if required) |
| Co-morbidities | Asthma (n) |
|  | Asthma (%) |
|  | Respiratory infectious disease (n) |
|  | Respiratory infectious disease (%) |
|  | Other infectious disease (n) |
|  | Other infectious disease (%) |
|  | Overweight (n) |
|  | Overweight (%) |
|  | Underweight (n) |
|  | Underweight (%) |
|  | Cystic fibrosis (n) |
|  | Cystic fibrosis (%) |
|  | Cardiomyopathy (n) |
|  | Cardiomyopathy (%) |
|  | Cardiac failure (n) |
|  | Cardiac failure (%) |
|  | Osteoporosis (n) |
|  | Osteoporosis (%) |
|  | Osteoporosis (linked to steroid use) (n) |
|  | Osteoporosis (linked to steroid use) (%) |
|  | Vit D Deficiency (n) |
|  | Vit D Deficiency (%) |
|  | Co-morbidity comments (if required) |
| General | Other EPI patient characteristics comments |
| *Note all Means require sds and medians require IQR (included in main extraction sheets)* | |

| **OUTCOMES** | |
| --- | --- |
| Study details | Endnote ID |
|  | First author and publication year |
|  | Country/countries (list separated by ";") |
|  | Population studied (DMD, Muscular dystrophy, DMD/BMD) |
|  | Subgroup (select one) |
| Epidemiology | Number of cases |
|  | Prevalence date |
|  | Prevalence per 100,000 |
|  | Lower CI prevalence per 100,000 |
|  | Upper CI prevalence per 100,000 |
|  | Definition for prevalence |
|  | Method for calculating prevalence |
|  | Comments on prevalence |
|  | Number of person years |
|  | Incidence per 1000 person years |
|  | Definition for incidence |
|  | Data collection period for incidence |
|  | Method for calculating incidence |
|  | Comments on incidence |
|  | Birth period |
|  | No births for study period |
| Mortality | Mean lifespan |
|  | Number of deaths |
|  | % overall mortality |
|  | SMR |
|  | SMR definition |
|  | Mortality comments (if required) |
| General | Reported limits of the study |
|  | Other EPI outcome comments |

| **GUIDELINES** | |
| --- | --- |
| Study details | Endnote ID |
|  | Study name |
|  | First author and publication year |
|  | Other related publications (list separated by ";") |
|  | Study group/organisation (if relevant) |
|  | World region |
|  | Country/countries (list separated by ";") |
|  | Guideline title |
|  | Brief description of scope |
|  | Audience type (select one) |
|  | Brief further details of audience type |
|  | Evidence grading system used (select one) |
|  | Brief further details of evidence grading used |
|  | Type of method for guideline development |
|  | Brief further details of guideline development process |
|  | Date for update (if reported) |
| Treatment Pathways | Does the guideline include a treatment pathway? |
| Standard of Care | Does the guideline include a definition of standard care? |
|  | Description of standard of care |
| Recommendations | Recommendation type (select one) |
|  | Evidence grade (if reported) |
|  | Treatment type |
|  | Further details of treatment type |
|  | Population type to which recommendation applies |
|  | Further details of population to which recommendation applies |
|  | Brief summary of recommendation |
| Overview | Other Guideline comments |

| **TREATMENT** | |
| --- | --- |
| Study details | Endnote ID |
|  | Study name |
|  | Country/countries (list separated by ";") |
| Treatment description | Treatment type (authors description) |
|  | Further details of intervention (format: Drug (dose) IV/oral frequency (use abbreviations)) |
|  | Number on treatment |
|  | % on treatment |
|  | Brief description of any other concomitant treatments or procedures given or allowed |
|  | Comments on treatment (if reqd) |
| Supportive treatment | Physiotherapy treatment (n) |
|  | Physiotherapy treatment (%) |
|  | Scoliosis surgery (n) |
|  | Scoliosis surgery (%) |
|  | Comments on supportive treatment (if reqd) |
| Overview | Other Treatment comments |
| *Note a new row is required for each treatment* | |

| **UTILITIES** | |
| --- | --- |
| Study details | Endnote ID |
|  | Study name |
|  | First author surname |
|  | Year |
|  | Other related publications (list separated by ";") |
|  | Study group/organisation (if relevant) |
|  | Country/countries (list separated by ";") |
| Study design & methodology | Study aim (authors aim) |
|  | Study design type (select one) |
|  | Further details of study design |
|  | Setting details (brief description) |
|  | Data collection years |
|  | Main data source type (select one) |
|  | Name main data source type if other |
|  | Data source name (e.g. database/survey name) |
|  | Further details of data source if required |
|  | Follow-up (mths) |
|  | Study conclusions (authors conclusions) |
| Population description | Type of study population (select one) |
|  | Whole population or subgroup (select one) |
|  | Further description of study population, if required |
|  | Total number of patients included in study (N) |
|  | Mean age (yrs) |
|  | Age SD (yrs) |
|  | Median age (yrs) |
|  | Lower IQR (yrs) |
|  | Upper IQR (yrs) |
|  | Min age (yrs) |
|  | Max age (yrs) |
|  | Number of males |
|  | % of males |
|  | Number of females |
|  | % of females |
|  | Overall ethnicity grouping (select one) |
|  | List of ethnic groups with % (format - White (25%); Asian (30%) etc. ) |
|  | Overall DMD severity related type - authors description |
|  | List of DMD severity-related groups with % (format - Mild (50%); moderate (27%); etc.) |
|  | Inclusion criteria (if reported, brief summary, separated by ";") |
|  | Exclusion criteria (if reported, brief summary, separated by ";") |
|  | Further description of at risk population, if required (brief summary) |
| Utility results (at baseline) | Method of elicitation (select one) |
|  | Method of elicitation if other |
|  | Further details of elicitation method, required |
|  | Indirect-HRQoL tool used (select one) |
|  | Indirect-HRQoL tool used if other |
|  | Further details of HRQoL used, if required |
|  | Treatment (authors description) |
|  | Mean utility/HRQoL |
|  | Lower 95% CI |
|  | Upper 95% CI |
| Utility results (at follow-up i.e. progression of disease) | Follow up Period (months) |
|  | Mean utility/HRQoL |
|  | Lower 95% CI |
|  | Upper 95% CI |
|  | utility/HRQoL (sd) |
|  | Mean change in utility/HRQoL |
|  | Mean Change in Lower 95% CI |
|  | Mean Change in Upper 95% CI |
|  | utility/HRQoL (sd) of Mean Change |
|  | Comments on follow up |
| COMMENTS | Overall Utility comments |

| **COSTS** | |
| --- | --- |
| Study details | Endnote ID |
|  | Study name |
|  | First author surname |
|  | Year |
|  | Other related publications (list separated by ";") |
|  | Study group/organisation (if relevant) |
|  | Country/countries (list separated by ";") |
| Study design & methodology | Study aim (authors aim) |
|  | Study design type (select one) |
|  | Further details of study design |
|  | Setting details (brief description) |
|  | Data collection years |
|  | Main data source type (select one) |
|  | Name main data source type if other |
|  | Cost Year |
|  | Currency |
|  | Discount rate (if used) |
|  | Data source name (e.g. database/survey name) |
|  | Further details of data source if required |
|  | Follow-up (mths) |
|  | Study conclusions (authors conclusions) |
|  | Design Comments |
| Population description | Type of study population (select one) |
|  | Whole population or subgroup (select one) |
|  | Further description of study population, if required |
|  | Total number of patients included in study (N) |
|  | Mean age (yrs) |
|  | Age SD (yrs) |
|  | Median age (yrs) |
|  | Lower IQR (yrs) |
|  | Upper IQR (yrs) |
|  | Min age (yrs) |
|  | Max age (yrs) |
|  | % of males |
|  | Number of females |
|  | % of females |
|  | Popn Comments |
| Direct Healthcare (units and cost per person) per annum | No. of admission /hospitalisation |
|  | Unit cost of admission /hospitalisation |
|  | SD cost of admission /hospitalisation |
|  | Total cost of admission /hospitalisation |
|  | No. of surgical procedures |
|  | Unit cost of surgical procedures |
|  | SD cost of surgical procedures |
|  | Total cost of surgical procedures |
|  | Type of medication |
|  | Unit cost of medication |
|  | SD cost of medication |
|  | Total cost of medication |
|  | Type of other direct costs |
|  | Unit cost of other direct costs |
|  | SD cost of other direct costs |
|  | Total cost of other direct costs |
| Indirect Healthcare (units and cost per person) per annum | Mean indirect (due to days off work) cost |
|  | SD indirect (due to days off work) cost |
|  | Calculation method (human capital or friction costs) |
|  | Days off work (mean) |
|  | Days off work (SD) |
|  | Sick-leave days (mean) |
|  | Sick-leave days (sd) |
|  | Sick-leave cost (mean) |
|  | Sick-leave cost (sd) |
|  | Early retirement/disability cost (mean) |
|  | Early retirement/disability cost (sd) |
|  | Reduced working time cost (mean) |
|  | Reduced working time cost (sd) |
|  | Change in type of work cost - (mean) |
|  | Change in type of work cost - (sd) |
|  | Other indirect cost type |
|  | Other indirect cost (mean) |
|  | Other indirect cost (sd) |
| Social care (units and cost per person) | Social care cost (mean) |
|  | Social care cost (sd) |
|  | Informal carer time (Mean days) |
|  | Informal carer time (sd days) |
|  | Care help cost (mean) |
|  | Care help cost (sd) |
|  | Equipment cost (mean) |
|  | Equipment cost (sd) |
|  | Other social care cost type |
|  | Other social care cost (mean) |
|  | Other social care cost (sd) |
| Out of Pocket Expenses (units and cost per person) | Patient / family cost (mean) |
|  | Patient / family cost (sd) |
|  | Other cost type |
|  | Cost of other categories (mean) |
|  | Cost of other categories (sd) |
| COMMENTS | Overall Cost comments |

| **QUALITY** | |
| --- | --- |
| Study identifier | Endnote ID |
|  | First author and publication year |
| 1 | Was there an adequate description of study design? |
| 2 | Was there an adequate description of eligibility criteria? |
| 3 | Is the study population representative of the target population? |
| 4 | Is there an adequate description of outcomes? |
| 5 | Is there an adequate description of the study participants? |
| Score | Overall score |
| Comments | Other Quality comments |
