# Supplementary material for: The burden, epidemiology, costs and treatment for Duchenne muscular dystrophy: an evidence review
Source: Orphanet J Rare Dis. 2017 Apr 26;12:79. doi: 10.1186/s13023-017-0631-3 (PMC5405509; doi:10.1186/s13023-017-0631-3)
Supplement: Supplementary file 4 — Study reporting quality (STROBE) and characteristics. (DOC 1545 kb) [file 13023_2017_631_MOESM4_ESM.doc]

# Additional file 4: Appendix 4: STUDY Reporting QUALITY (STROBE) AND CHARACTERISTICS

Table A1: Quality of birth prevalence data in DMD patients

| **First author & publication year** | **1. Was there an adequate description of study design?** | **2. Was there an adequate description of eligibility criteria?** | **3. Is the study population representative of the target population?** | **4. Is there an adequate description of outcomes?** | **5. Is there an adequate description of the study participants?** | **Overall assessment** |
| --- | --- | --- | --- | --- | --- | --- |
| Moat, 2013[^3^](#_ENREF_3) | Yes | No | Yes | Yes | No | Medium |
| Mendell, 2012[^2^](#_ENREF_2) | Yes | Yes | Yes | Yes | No | Medium |

Table A2: Quality of prevalence studies

| **First author & publication year** | **1. Was there an adequate description of study design?** | **2. Was there an adequate description of eligibility criteria?** | **3. Is the study population representative of the target population?** | **4. Is there an adequate description of outcomes?** | **5. Is there an adequate description of the study participants?** | **Overall assessment** |
| --- | --- | --- | --- | --- | --- | --- |
| Norwood FL, 2009[^12^](#_ENREF_12) | Yes | Yes | Yes | Yes | No | Medium |
| Rasmussen, 2012[^13^](#_ENREF_13) | Yes | NR/unclear | Yes | Yes | No | Medium |
| Romitti, 2015[^14^](#_ENREF_14) | No | No | Yes | Yes | No | Low |
| Mah, 2011[^15^](#_ENREF_15) | Yes | No | No | No | No | Low |
| Bladen, 2015[^65^](#_ENREF_65) | Yes | No | No | Yes | No | Low |

Table A3: Quality of studies reporting mortality data in DMD patients

| **First author & publication year** | **1. Was there an adequate description of study design?** | **2. Was there an adequate description of eligibility criteria?** | **3. Is the study population representative of the target population?** | **4. Is there an adequate description of outcomes?** | **5. Is there an adequate description of the study participants?** | **Overall assessment** |
| --- | --- | --- | --- | --- | --- | --- |
| Kieny, 2013[^17^](#_ENREF_17) | Yes | Yes | Yes | Yes | No | Medium |
| Passamano, 2012[^18^](#_ENREF_18) | No | Yes | NR/unclear | No | No | Low |
| Rall, 2012[^19^](#_ENREF_19) | Yes | Yes | Yes | Yes | No | Medium |

Table A4: Study quality for severity and progression studies

| **First author & publication year** | **1. Was there an adequate description of study design?** | **2. Was there an adequate description of eligibility criteria?** | **3. Is the study population representative of the target population?** | **4. Is there an adequate description of outcomes?** | **5. Is there an adequate description of the study participants?** | **Overall assessment** |
| --- | --- | --- | --- | --- | --- | --- |
| Ashwath, 2014[^37^](#_ENREF_37) | Yes | No | NR/unclear | Yes | No | Low |
| Bello, 2015[^25^](#_ENREF_25) | Yes | NR/unclear | Yes | Yes | No | Medium |
| Bladen, 2015[^65^](#_ENREF_65) | Yes | No | No | Yes | No | Low |
| Connolly, 2013[^41^](#_ENREF_41) | NR/unclear | NR/unclear | NR/unclear | NR/unclear | No | Low |
| Davidson, 2014[^5^](#_ENREF_5) | No | No | NR/unclear | Yes | No | Low |
| de Moura, 2015[^29^](#_ENREF_29) | Yes | Yes | NR/unclear | Yes | No | Medium |
| Fox, 2015[^48^](#_ENREF_48) | Yes | Yes | Yes | Yes | No | Medium |
| Henricson, 2012[^66^](#_ENREF_66) | Yes | NR/unclear | Yes | Yes | NR/unclear | Medium |
| Henricson, 2013[^21^](#_ENREF_21) | Yes | Yes | Yes | Yes | Yes | High |
| Janssen, 2014[^36^](#_ENREF_36) | Yes | Yes | NR/unclear | Yes | No | Medium |
| Kempen, 2014[^67^](#_ENREF_67) | NR/unclear | Yes | Yes | NR/unclear | No | Low |
| Khirani, 2014[^35^](#_ENREF_35) | Yes | Yes | NR/unclear | No | No | Low |
| Kieny, 2012[^17^](#_ENREF_17) | Yes | Yes | Yes | Yes | No | Medium |
| Larkindale, 2014[^32^](#_ENREF_32) | Yes | No | No | Yes | No | Low |
| Lerario, 2012[^68^](#_ENREF_68) | NR/unclear | Yes | NR/unclear | NR/unclear | No | Low |
| Lorusso, 2013[^31^](#_ENREF_31) | Yes | Yes | NR/unclear | Yes | No | Medium |
| Magri, 2011[^4^](#_ENREF_4) | Yes | NR/unclear | Yes | No | No | Low |
| Magri, 2011[^30^](#_ENREF_30) | Yes | NR/unclear | Yes | Yes | No | Medium |
| Mah, 2012[^44^](#_ENREF_44) | NR/unclear | NR/unclear | NR/unclear | NR/unclear | No | Low |
| Mah,2011[^15^](#_ENREF_15) | Yes | No | No | No | No | Low |
| Martigne, 2011[^28^](#_ENREF_28) | Yes | NR/unclear | Yes | Yes | No | Medium |
| Mayer, 2015[^27^](#_ENREF_27) | Yes | Yes | Yes | No | NR/unclear | Medium |
| Mazzone,2014[^46^](#_ENREF_46) | Yes | Yes | NR/unclear | Yes | No | Medium |
| Mazzone,2014[^74^](#_ENREF_74) | Yes | NR/unclear | Yes | Yes | No | Medium |
| McDonald, 2013[^22^](#_ENREF_22) | Yes | Yes | Yes | Yes | Yes | High |
| Nakamura, 2013[^26^](#_ENREF_26) | Yes | Yes | Yes | NR/unclear | No | Medium |
| Pane, 2014[^34^](#_ENREF_34) | Yes | Yes | Yes | Yes | No | Medium |
| Pane, 2014[^42^](#_ENREF_42) | Yes | Yes | Yes | Yes | No | Medium |
| Pane, 2014[^75^](#_ENREF_75) | Yes | Yes | NR/unclear | NR/unclear | No | Low |
| Passamano, 2012[^18^](#_ENREF_18) | No | Yes | NR/unclear | No | No | Low |
| Rall, 2012[^19^](#_ENREF_19) | Yes | Yes | Yes | Yes | No | Medium |
| Ricotti, 2012[^47^](#_ENREF_47) | Yes | NR/unclear | NR/unclear | NR/unclear | No | Low |
| Roberto, 2011[^70^](#_ENREF_70) | Yes | NR/unclear | Yes | Yes | No | Medium |
| Rodger, 2015[^39^](#_ENREF_39) | Yes | No | NR/unclear | No | No | Low |
| Schreiber-Katz, 2014[^23^](#_ENREF_23) | Yes | Yes | Yes | Yes | Yes | High |
| Seferian, 2015[^72^](#_ENREF_72) | No | Yes | Yes | No | NR/unclear | Medium |
| Soderpalm, 2012[^45^](#_ENREF_45) | Yes | No | Yes | Yes | No | Medium |
| Spurney, 2014[^24^](#_ENREF_24) | Yes | No | NR/unclear | Yes | No | Low |
| Thomas, 2012[^38^](#_ENREF_38) | Yes | NR/unclear | NR/unclear | Yes | No | Low |
| Vry, 2013[^40^](#_ENREF_40) | NR/unclear | NR/unclear | NR/unclear | NR/unclear | No | Low |
| West, 2013[^73^](#_ENREF_73) | Yes | Yes | Yes | NR/unclear | No | Medium |

Table A5: Quality of studies reporting treatment for DMD patients

| **First author & publication year** | **1. Was there an adequate description of study design?** | **2. Was there an adequate description of eligibility criteria?** | **3. Is the study population representative of the target population?** | **4. Is there an adequate description of outcomes?** | **5. Is there an adequate description of the study participants?** | **Overall assessment** |
| --- | --- | --- | --- | --- | --- | --- |
| Bello(a), 2015[^25^](#_ENREF_25) | Yes | NR/unclear | Yes | Yes | No | Medium |
| Fox, 2015[^48^](#_ENREF_48) | Yes | Yes | Yes | Yes | No | Medium |
| Henricson, 2012[^66^](#_ENREF_66) | Yes | NR/unclear | Yes | Yes | NR/unclear | Medium |
| Janssen, 2014[^36^](#_ENREF_36) | Yes | Yes | NR/unclear | Yes | No | Medium |
| Khirani, 2014[^35^](#_ENREF_35) | Yes | Yes | NR/unclear | No | No | Low |
| Kieny, 2013[^17^](#_ENREF_17) | Yes | Yes | Yes | Yes | No | Medium |
| Magri, 2011(a)[^4^](#_ENREF_4) | Yes | NR/unclear | Yes | No | No | Low |
| Mayer, 2015[^27^](#_ENREF_27) | Yes | Yes | Yes | No | NR/unclear | Medium |
| Pane(a), 2014[^34^](#_ENREF_34)} | Yes | Yes | Yes | Yes | No | Medium |
| McDonald(e), 2013[^69^](#_ENREF_69) | Yes | Yes | Yes | No | No | Medium |
| Nakamura, 2013[^26^](#_ENREF_26) | Yes | Yes | Yes | NR/unclear | No | Medium |
| Ricotti, 2012[^47^](#_ENREF_47) | Yes | NR/unclear | NR/unclear | NR/unclear | No | Low |
| Roberto, 2011[^70^](#_ENREF_70) | Yes | NR/unclear | Yes | Yes | No | Medium |
| Rodger, 2015[^39^](#_ENREF_39) | Yes | No | NR/unclear | No | No | Low |
| Sarrazin, 2014[^71^](#_ENREF_71) | Yes | Yes | Yes | NR/unclear | No | Medium |
| Schreiber-Katz, 2014[^23^](#_ENREF_23) | Yes | Yes | Yes | Yes | Yes | High |
| Soderpalm, 2012[^45^](#_ENREF_45) | Yes | No | Yes | Yes | No | Medium |
| Vry, 2013[^40^](#_ENREF_40) | NR/unclear | NR/unclear | NR/unclear | NR/unclear | No | Low |

Table A6: Quality of studies reporting HRQoL / utility for DMD patients

| **First author & publication year** | **1. Was there an adequate description of study design?** | **2. Was there an adequate description of eligibility criteria?** | **3. Is the study population representative of the target population?** | **4. Is there an adequate description of outcomes?** | **5. Is there an adequate description of the study participants?** | **Overall assessment** |
| --- | --- | --- | --- | --- | --- | --- |
| Bendixen, 2014[^51^](#_ENREF_51) | Yes | NR/unclear | Yes | NR/unclear | No | Low |
| de Moura, 2015[^29^](#_ENREF_29) | Yes | Yes | NR/unclear | Yes | No | Medium |
| Henricson, 2013[^21^](#_ENREF_21) | Yes | Yes | Yes | Yes | Yes | High |
| Houwen-van Opstal, 2014[^57^](#_ENREF_57) | Yes | NR/unclear | Yes | No | Yes | Medium |
| Landfeldt(a), 2014[^58^](#_ENREF_58) | Yes | Yes | Yes | Yes | NR/unclear | Medium |
| Lim, 2014[^50^](#_ENREF_50) | Yes | No | NR/unclear | Yes | No | Low |
| Schreiber-Katz, 2014[^23^](#_ENREF_23) | Yes | Yes | Yes | Yes | Yes | High |
| Pentek, 2014[^49^](#_ENREF_49) | Yes | No | NR/unclear | No | No | Low |
| Pangalila(a), 2015[^54^](#_ENREF_54) | Yes | Yes | NR/unclear | Yes | No | Medium |
| Simon, 2011[^55^](#_ENREF_55) | Yes | No | No | Yes | Yes | Medium |
| Baiardini, 2011[^56^](#_ENREF_56) | Yes | No | No | Yes | Yes | Medium |
| Bendixen, 2012[^53^](#_ENREF_53) | Yes | No | NR/unclear | Yes | Yes | Medium |
| Uzark, 2012[^52^](#_ENREF_52) | Yes | Yes | No | Yes | No | Medium |

Table A7: Quality of studies reporting cost of illness for DMD patients

| **First author & publication year** | **1. Was there an adequate description of study design?** | **2. Was there an adequate description of eligibility criteria?** | **3. Is the study population representative of the target population?** | **4. Is there an adequate description of outcomes?** | **5. Is there an adequate description of the study participants?** | **Overall assessment** |
| --- | --- | --- | --- | --- | --- | --- |
| Landfeldt(a), 2014[^58^](#_ENREF_58) | Yes | Yes | Yes | Yes | NR/unclear | Medium |
| Larkindale, 2014[^32^](#_ENREF_32) | Yes | No | No | Yes | No | Low |
| Schreiber-Katz, 2014[^23^](#_ENREF_23) | Yes | Yes | Yes | Yes | Yes | High |

Table A8: Characteristics of prevalence studies derived from literature searches

| **Reference** | **Data collection period** | **Study design** | **Study duration (years)** | **Country** | **Region** | **Population of interest** | **Inclusion criteria** | **Study Conclusions** |
| --- | --- | --- | --- | --- | --- | --- | --- | --- |
| Mendell, 2012[^2^](#_ENREF_2) | 03/2007 to 01/2011 | Newborn screening | 3 | USA | Ohio | Newborn males | Phase 1 dried blood spot samples from anonymous newborns within 48 hrs, max 120 hrs; phase 2 and 3 - newborns of parents who consented within 48 hrs; phase 4 - de-identified samples | A 2-tier system of analysis for newborn screening minimizes false-positives and uses predetermined levels of CK on dried blood spots to predict DMD gene mutations. |
| Moat, 2013[^3^](#_ENREF_3) | 01/1990 to 12/2011 | Newborn screening | 21 | UK | Wales | Newborn males | Newborn screening blood spot cards from Welsh boys; tested for blood spot CK following parental consent. | Screening has enabled reproductive choice for parents of affected boys and earlier therapy |
| Norwood FL, 2009[^12^](#_ENREF_12) | 08/2007 | Cross-sectional | NA | UK | Northern England | Males with inherited muscle disease | All registered patients with inherited muscle diseases diagnosed and seen by the neuromuscular team at the Institute of Human Genetics | The study illustrates the immense diagnostic progress since the first regional survey over 50 years ago. |
| Rasmussen, 2012[^13^](#_ENREF_13) | 08/2015 | Cross-sectional | NA | Norway | SE Norway | Males < 18 with neuromuscular disease | Known/suspected neuromuscular disorder was confirmed following diagnostic work up at Rikshospitalet University Hospital | DMD was the largest group of neuromuscular disorders but it was difficult to make specific diagnoses in quite a few cases. |
| Romitti, 2015[^14^](#_ENREF_14) | 01/1982 to 12/2011 | Cross-sectional | 30 | US | NR | Males 5 to 9 born between 01/1982 and 11/2011 with childhood onset Duchene Becker Muscular dystrophy | Males born between 01/1982 and 11/2011, residing in MD Starnet site with childhood onset Duchenne Becker Muscular Dystrophy | Prevalence differed by ethnicity, suggesting potential cultural and socioeconomic influences in the diagnosis of DBMD. Prevalence also was higher for DMD than BMD. |
| Mah, 2011[^15^](#_ENREF_15) | 01/2000 to 12/2009 | Cross-sectional | 10 | Canada | NR | Males 0 to 24 | Molecular genetic reports from DBMD patients followed by participating CPNG centres from January 2000 to December 2009 | Consensus guidelines will hopefully reduce the geographical variation in mutation detection rates in the coming decade |
| Bladen 2013[^16^](#_ENREF_16) | 2007 to 2012 | Worldwide network of registries | 5 | Worldwide | NR | DMD patients | DMD registry members in August 2012 collected via a comprehensive  Questionnaire. | Global and national DMD patient registries provide an unparalleled resource for patient information, clinical and academic research, and best standards of care assurance. |

NA, not available; NR, not reported

Table A9: Characteristics of mortality studies

| **First author & publication year** | **Data collection period** | **Study design** | **Study duration (years)** | **Country** | **Population of interest** | **Inclusion criteria** | **Study Conclusions** |
| --- | --- | --- | --- | --- | --- | --- | --- |
| Kieny, 2013 | 01/1981 to 09/2011 | retrospective cohort | 30 | France | Adult DMD patients | All temporary or permanent adult residents labelled DMD at AFM Yolaine de Kepper centre between 1981 and September 2011 were included | Ventilator assistance, mostly through tracheotomy, prolongs life by more than 15 years for DMD patients. It allows conservation of a satisfactory quality of life, and should be systematically proposed to patients. |
| Passamano, 2012[^18^](#_ENREF_18) | 1961 to 2006 | retrospective cohort | 46 | Italy | All age | DMD patients; followed at inclusion centre in inclusion period; follow up > 25 years | DMD should be now considered an adulthood disease and as a consequence more public health interventions are needed to support these patients and their families as they pass from childhood into adult age. |
| Rall, 2012[^19^](#_ENREF_19) | 2009 | retrospective cohort | 39 | Germany | All DMD | Born 1970 - 1980 with proven out-of-frame mutation or when “registered as a DMD patient”. | The study provides survival data for a cohort of DMD patients in Germany stratified by year of death. Median survival was 24.0 years in patients confirmed by molecular testing. |

Table A10: Study characteristics for studies of severity and progression

| **First author & publication year** | **Country/countries** | **Data collection period** | **Name of subgroup** | **Sample size** |
| --- | --- | --- | --- | --- |
| Ashwath, 2014[^37^](#_ENREF_37) | US | 1999 to 2011 | All DMD | 75 |
| Bello, 2015[^25^](#_ENREF_25) | Worldwide | NR |  | 340 |
|  |  |  | Ambulatory | 111 |
|  |  |  | Non-ambulatory | 229 |
| Bladen, 2015[^65^](#_ENREF_65) |  |  | All DMD mutations | NR |
| Connolly, 2013[^41^](#_ENREF_41) | US |  | Boys <3y | 24 |
| Davidson, 2014[^5^](#_ENREF_5) | Australia |  | All DMD | 144 |
| de Moura, 2015[^29^](#_ENREF_29) | Brazil |  |  | 31 |
|  |  |  |  | 34 |
| Fox, 2015[^48^](#_ENREF_48) | US | 1982 to 2001 |  | 521 |
|  |  |  | Any steroid use | 310 |
|  |  |  | No steroid use | 211 |
| Henricson, 2012[^66^](#_ENREF_66) |  | NR | Ambulatory-boys | 17 |
| Henricson, 2013[^21^](#_ENREF_21) |  |  |  | 24 |
| Janssen, 2014[^36^](#_ENREF_36) | Italy; the Netherlands;England;Spain;USA;Germany;Belgium;Switzerland;Canada;Ireland;Australia;Nepal;Peru;India |  | All DMD | 213 |
|  |  |  | Early ambulatory stage | 66 |
|  |  |  | Early non-ambulatory stage | 24 |
|  |  |  | Late ambulatory stage | 29 |
|  |  |  | Late non-ambulatory stage | 94 |
| Kempen, 2014[^67^](#_ENREF_67) | Netherlands |  | Ambulatory >150m with or without walking aid, ≥6y | 19 |
| Khirani, 2014[^35^](#_ENREF_35) | France | 2001 to 2011 | All DMD | 48 |
| Kieny, 2013[^17^](#_ENREF_17) |  | 1981 to 2011 | all DMD-adults | 119 |
|  |  |  | Born 1970-1980 - adults | 53 |
|  |  |  | Born after 1980 - adults | 23 |
|  |  |  | Born before 1970 - adults | 43 |
| Larkindale, 2014[^32^](#_ENREF_32) | US | 2008 to 2010 | All DMD | 95 |
| Lerario, 2012[^68^](#_ENREF_68) | Italy | NR | Ambulatory-boys | 28 |
| Lorusso, 2013[^31^](#_ENREF_31) |  |  | Boys 6y-12y | 42 |
| Magri, 2011[^4^](#_ENREF_4) |  |  | All DMD | 205 |
| Magri, 2011[^30^](#_ENREF_30) |  |  |  | 41 |
| Mah, 2012[^44^](#_ENREF_44) | NR |  | Boys | 340 |
| Mah,2011[^15^](#_ENREF_15) | Canada | 2000 to 2009 | All DMD | 529 |
| Martigne, 2011[^28^](#_ENREF_28) | France | NR |  | 70 |
| Mayer, 2015[^27^](#_ENREF_27) | US | 2005 to 2010 | >22y | 3 |
|  |  |  | 10-12y | 20 |
|  |  |  | 12-14y | 13 |
|  |  |  | 14-16y | 11 |
|  |  |  | 16-18y | 9 |
|  |  |  | 18-20y | 6 |
|  |  |  | 20-22y | 4 |
|  |  |  | 6-8y | 15 |
|  |  |  | 8-10y | 17 |
|  |  |  | <6y | 4 |
| Mazzone,2014[^46^](#_ENREF_46) | Italy | 2008 to 2011 | Ambulatory-boys | 113 |
| Mazzone,2014[^74^](#_ENREF_74) |  | 2008 to 2008 |  | 106 |
|  |  |  | Ambulatory <=7y, Continuous steroids | NR |
|  |  |  | Ambulatory boys <=7y | 35 |
|  |  |  | Ambulatory boys <=7y, None or intermittent steroids | NR |
|  |  |  | Ambulatory boys >7y | 71 |
|  |  |  | Ambulatory boys >7y, Continuous steroids | NR |
|  |  |  | Ambulatory boys >7y, None or intermittent steroids | NR |
|  |  |  | Ambulatory boys-Continuous steroids | 55 |
|  |  |  | Ambulatory boys-None or intermittent steroids | 51 |
| McDonald, 2013[^22^](#_ENREF_22) | Australia; Belgium; Canada; France; Germany; Italy; Israel; Spain; Sweden; UK; USA | NR | Ambulatory >=5y | 57 |
|  |  |  | Ambulatory-Steroid treated, <7y | 6 |
|  |  |  | Ambulatory-Steroid treated, >=7y | 34 |
|  |  |  | Ambulatory-Steroid-naïve, <7y | 8 |
| McDonald, 2013[^69^](#_ENREF_69) | Argentina; Australia; Canada; India; Israel; Italy; Sweden; United States | 2006 to 2009 | All DMD | 340 |
| Mendell, 2012[^2^](#_ENREF_2) | US | 2007 to 2011 |  | 6 |
| Moat, 2013[^3^](#_ENREF_3) | UK | 1990 to 2011 |  | 72 |
| Nakamura, 2013[^26^](#_ENREF_26) | Japan | 2009 to 2012 |  | 583 |
| Norwood FL, 2009[^12^](#_ENREF_12) | UK | 2007 |  | 124 |
| Pane, 2014[^34^](#_ENREF_34) | Italy | 2008 to 2010 | Ambulatory-boys | 96 |
|  |  |  | Ambulatory <350m(6MWT), <7y | 9 |
|  |  |  | Ambulatory <350m(6MWT), ≥7y | 25 |
|  |  |  | Ambulatory ≥350m(6MWT), <7y | 19 |
|  |  |  | Ambulatory ≥350m(6MWT), ≥7y | 43 |
| Pane, 2014[^42^](#_ENREF_42) | Italy; Belgium | 2008 to NR | Ambulatory ≥100m | 191 |
|  |  |  | Ambulatory ≥100m, <7y | 80 |
|  |  |  | Ambulatory ≥100m, >7y | 111 |
|  |  |  | Ambulatory ≥100m-All deletions | 132 |
|  |  |  | Ambulatory ≥100m-Deletions eligible for skipping exon 44 | 18 |
|  |  |  | Ambulatory ≥100m-Deletions eligible for skipping exon 45 | 15 |
|  |  |  | Ambulatory ≥100m-Deletions eligible for skipping exon 46 | 7 |
|  |  |  | Ambulatory ≥100m-Deletions eligible for skipping exon 50 | 9 |
|  |  |  | Ambulatory ≥100m-Deletions eligible for skipping exon 51 | 27 |
|  |  |  | Ambulatory ≥100m-Deletions eligible for skipping exon 53 | 28 |
|  |  |  | Ambulatory ≥100m-Duplications | 15 |
|  |  |  | Ambulatory ≥100m-Point mutations | 44 |
| Pane, 2014[^75^](#_ENREF_75) | Italy | 2012-2014 | Ambulatory | 164 |
| Passamano, 2012[^18^](#_ENREF_18) |  | 1961 to 2006 | All DMD | 516 |
| Rall, 2012[^19^](#_ENREF_19) | Germany | 2009 | Confirmed molecular diagnosis | 67 |
| Ricotti, 2012[^47^](#_ENREF_47) | UK | 2004 to 2011 | Boys | 400 |
| Roberto, 2011[^70^](#_ENREF_70) | US | 1992 to 2007 | All DMD | 43 |
| Rodger, 2015[^39^](#_ENREF_39) | Bulgaria | 2011 to 2012 | Adults, non ambulatory | 7 |
|  |  |  | DMD - children | 33 |
|  | Bulgaria; the Czech Republic; Denmark; Germany; Hungary; Poland; the United Kingdom |  | Adults, non ambulatory | 201 |
|  | Denmark |  |  | 43 |
|  |  |  | DMD - children | 45 |
|  | Eastern Europe |  | Adults, non ambulatory | 39 |
|  |  |  | DMD - children | 289 |
|  | Germany |  | Adults, non ambulatory | 77 |
|  |  |  | DMD - children | 343 |
|  | Hungary |  | Adults, non ambulatory | 5 |
|  |  |  | DMD - children | 52 |
|  | Poland |  | Adults, non ambulatory | 16 |
|  |  |  | DMD - children | 126 |
|  | the Czech Republic |  | Adults, non ambulatory | 11 |
|  |  |  | DMD - children | 78 |
|  | UK |  | Adults, non ambulatory | 42 |
|  |  |  | DMD - children | 184 |
| Romitti, 2015[^14^](#_ENREF_14) | US | 1982 to 2011 | All DMD | 845 |
| Sarrazin, 2014[^71^](#_ENREF_71) | Germany | 1975 to 2011 |  | 263 |
| Schreiber-Katz, 2014[^23^](#_ENREF_23) |  | 2013 |  | 248 |
|  |  |  | Stage 1 DMD patient | 49 |
|  |  |  | Stage 2 DMD patient | 70 |
|  |  |  | Stage 3 DMD patient | 11 |
|  |  |  | Stage 4 DMD patient | 92 |
|  |  |  | Stage 5 DMD patient | 26 |
| Seferian, 2015[^72^](#_ENREF_72) | France | 2010 to 2013 | Non-ambulatory | 53 |
| Soderpalm, 2012[^45^](#_ENREF_45) | Sweden | 2003 to 2004 | <10y | 8 |
|  |  |  | >10y | 10 |
|  |  |  | All DMD | 18 |
| Spurney, 2014[^24^](#_ENREF_24) | US | 2006 to 2009 |  | 174 |
| Thomas, 2012[^38^](#_ENREF_38) |  | 2000 to 2009 | Boys <10y | 24 |
|  |  |  | Boys >=10y | 31 |
| Vry, 2013[^40^](#_ENREF_40) | Bulgaria; the Czech Republic; Denmark; Germany; Hungary; Poland; and the United Kingdom | NR | All DMD | 1071 |
| West, 2013[^73^](#_ENREF_73) | US | 1985 to 2010 | 10 to 10.49y | 45 |
|  |  |  | 10.5 to 10.99y | 40 |
|  |  |  | 11 to 11.49y | 29 |
|  |  |  | 11.5 to 11.99y | 29 |
|  |  |  | 2 to 2.49y | 76 |
|  |  |  | 2.5 to 2.99y | 87 |
|  |  |  | 3 to 3.49y | 108 |
|  |  |  | 3.5 to 3.99y | 111 |
|  |  |  | 4 to 4.49y | 136 |
|  |  |  | 4.5 to 4.99y | 145 |
|  |  |  | 5 to 5.49y | 154 |
|  |  |  | 5.5 to 5.99y | 144 |
|  |  |  | 6 to 6.49y | 149 |
|  |  |  | 6.5 to 6.99y | 121 |
|  |  |  | 7 to 7.49y | 111 |
|  |  |  | 7.5 to 7.99y | 103 |
|  |  |  | 8 to 8.49y | 92 |
|  |  |  | 8.5 to 8.99y | 80 |
|  |  |  | 9 to 9.49y | 63 |
|  |  |  | 9.5 to 9.99y | 54 |

Table A11: General severity

| **Country /countries** | **Name of subgroup** | **First author & publication year** | **n** | **Mean age** | **List of severity groups with %** |
| --- | --- | --- | --- | --- | --- |
| Germany | All DMD | Schreiber-Katz, 2014[^23^](#_ENREF_23) | 248 | NR | (Severity1)=19.8%;(Severity2)=28.2%;(Severity3)=4.4%;(Severity4)=37.1%;(Severity5)=10.5% |
| US | All DMD | Spurney, 2014[^24^](#_ENREF_24) | 174 | 12 | Early ambulatory 23.5%;late ambulatory 26.5%, early non ambulatory 14.2%;late non ambulatory 35.8% |

Table A12: Characteristics of drug therapy (corticosteroid) studies

| **DMD Grouping** | **DMD Sub group** | **Country** | **Date** | **First author & publication year** |
| --- | --- | --- | --- | --- |
| General DMD | All DMD | Bulgaria; Czech Republic; Denmark; Germany; Hungary; Poland; UK | NR | Vry, 2013[^40^](#_ENREF_40) |
|  |  | Germany | 2013 | Schreiber-Katz, 2014[^23^](#_ENREF_23) |
|  |  |  | 1975 to 2011 | Sarrazin, 2014[^71^](#_ENREF_71) |
|  |  | Italy; the Netherlands; England; Spain; USA; Germany; Belgium; Switzerland; Canada; Ireland; Australia; Nepal; Peru; India | NR | Janssen, 2014[^36^](#_ENREF_36) |
|  |  | Japan | 2009 to 2012 | Nakamura, 2013[^26^](#_ENREF_26) |
|  |  | Sweden | 2003 to 2004 | Soderpalm, 2012[^45^](#_ENREF_45) |
|  |  | USA | 1982 to 2001 | Fox, 2015[^48^](#_ENREF_48) |
|  |  | Worldwide | NR | Bello(a), 2015[^25^](#_ENREF_25) |
|  | All DMD at first visit | Argentina; Australia; Canada; India; Israel; Italy; Sweden; USA | 2006 to 2009 | McDonald(e), 2013[^69^](#_ENREF_69) |
|  |  | USA | 2005 to 2010 | Mayer, 2015[^27^](#_ENREF_27) |
|  | Diagnosis after 1995 | Italy | NR | Magri, 2011(a)[^4^](#_ENREF_4) |
|  | Diagnosis before 1995 | Italy | NR |  |
|  | Hispanic | US | 1982 to 2001 | Fox, 2015[^48^](#_ENREF_48) |
|  | Non-Hispanic Black | US | 1982 to 2001 |  |
|  | Non-Hispanic White | US | 1982 to 2001 |  |
| Ambulatory status | Ambulatory | Worldwide | NR | Bello(a), 2015[^25^](#_ENREF_25) |
|  | Early ambulatory stage | Italy; the Netherlands; England; Spain; USA; Germany; Belgium; Switzerland; Canada; Ireland; Australia; Nepal; Peru; India | NR | Janssen, 2014[^36^](#_ENREF_36) |
|  | Early non-ambulatory stage |  | NR |  |
|  | Late ambulatory stage |  | NR |  |
|  | Late non-ambulatory stage |  | NR |  |
|  | Non ambulatory | Worldwide | NR | Bello(a), 2015[^25^](#_ENREF_25) |
|  | Ambulant - boys | Italy | 2008 to 2008 | Pane(a), 2014[^34^](#_ENREF_34), Mazzone, 2011[^74^](#_ENREF_74) |
|  |  | UK | 2004 to 2011 | Ricotti, 2012[^47^](#_ENREF_47) |
|  |  | US | NR | Henricson, 2012[^66^](#_ENREF_66) |

Table A13: Study characteristics, HRQoL studies

| **First author & publication year** | **Country /countries** | **Data collection years** | **HRQoL tool** | **Name of subgroup** | **Sample size** |
| --- | --- | --- | --- | --- | --- |
| Baiardini, 2011[^56^](#_ENREF_56) | Italy | NR | Children Health Questionnaire - Parent Form 50 | Italian DMD Boys | 27 |
| Bendixen, 2012[^53^](#_ENREF_53) | US |  | PedsQL | Boys < 10y | 27 |
|  |  |  |  | Boys >= 10y | 23 |
| Bendixen, 2014[^51^](#_ENREF_51) | US; Canada | 2009 to 2013 | CAPE Physical activity domain | NR | 60 |
|  |  |  | CAPE Recreational activity domain |  | 60 |
|  |  |  | CAPE Social activity domain |  | 60 |
|  |  |  | CAPE Skill-based activity domain |  | 60 |
|  |  |  | CAPE Self-improvement activity domain |  | 60 |
|  |  |  | CAPE With whom activity dimension |  | 60 |
|  |  |  | CAPE Where activity dimension |  | 60 |
|  |  |  | CAPE Enjoyment activity dimension |  | 60 |
|  |  |  | CAPE | Age <10y | 35 |
|  |  |  |  | Age>=10 | 25 |
| de Moura, 2015[^29^](#_ENREF_29) | Brazil | NR | Autoquestionnaire Qualité de vie Enfant Imagé (AUQEI) | All DMD | 34 |
| Henricson, 2013[^21^](#_ENREF_21) | US |  | PedsQL | Boys-ambulatory | 24 |
|  |  |  | PODCI |  | 24 |
| Houwen-van Opstal, 2014[^57^](#_ENREF_57) | Netherlands |  | KIDSCREEN-52 physical domain | Ambulant | 19 |
|  |  |  |  | Non-ambulant, decreased arm abilities | 7 |
|  |  |  |  | Non-ambulant, relatively good arm abilities | 14 |
|  |  |  |  | NR | 40 |
| Lim, 2014[^50^](#_ENREF_50) |  | NR | PedsQL | Boys | 63 |
|  |  |  |  | Boys Parents as proxy | 63 |
| Pangalila, 2015[^54^](#_ENREF_54) | Netherlands |  | SF-36 | Adult DMD | 79 |
|  |  |  | WHOQOL-BREF |  | 79 |
|  |  |  | Fatigue Severity Score |  | 79 |
|  |  |  | HADS |  | 79 |
| Pentek, 2014[^49^](#_ENREF_49) | Hungary |  | Barthel Index | All DMD | 57 |
| Schreiber-Katz, 2014[^23^](#_ENREF_23) | Germany | 2013 | PedsQL |  | 248 |
|  |  |  |  | Parent as proxy | 248 |
|  |  |  |  | Stage 1 Parent as proxy | 49 |
|  |  |  |  | Stage 2 Parent as proxy | 70 |
|  |  |  |  | Stage 3 Parent as proxy | 11 |
|  |  |  |  | Stage 4 Parent as proxy | 92 |
|  |  |  |  | Stage 5 Parent as proxy | 26 |
|  |  |  |  | Stage 1 DMD | 49 |
|  |  |  |  | Stage 2 DMD | 70 |
|  |  |  |  | Stage 3 DMD | 11 |
|  |  |  |  | Stage 4 DMD | 92 |
|  |  |  |  | Stage 5 DMD | 26 |
| Simon, 2011[^55^](#_ENREF_55) | Brazil | 2007 to 2008 | Life Satisfaction Index for Adolescents (LSI-A) | Age 11-13 | 28 |
|  |  |  |  | Age 13-17 | 16 |
|  |  |  |  | Age 5-7 | 11 |
|  |  |  |  | Age 8-10 | 40 |
| Uzark, 2012[^52^](#_ENREF_52) | US | NR | PedsQL | Age 13-18 | 46 |
|  |  |  |  | Age 5-18 | 203 |
|  |  |  |  | Age 8-12 | 106 |
|  |  |  |  | Age 5-7 Parent as proxy | 51 |
|  |  |  |  | Age 8-12 Parent as proxy | 106 |
|  |  |  |  | Age 13-18 Parent as proxy | 46 |

Table A14: Study characteristics and results, utility studies

| **First author & publication year** | **Country /countries** | **Data collection years** | **Utility tool** | **Name of subgroup** | **Sample size** | **Mean utility** | **Utility (sd)** |
| --- | --- | --- | --- | --- | --- | --- | --- |
| Landfeldt, 2014[^58^](#_ENREF_58) | Germany | 2012 to 2013 | HUI | German DMD Boys | 173 | 0.45 | NR |
|  |  |  |  | German DMD Boys-early ambulatory | 30 | 0.8 |  |
|  |  |  |  | German DMD Boys-early non ambulatory | 47 | 0.27 |  |
|  |  |  |  | German DMD Boys-late ambulatory | 49 | 0.73 |  |
|  |  |  |  | German DMD Boys-late non ambulatory | 47 | 0.13 |  |
|  | Italy |  |  | Italian DMD Boys | 122 | 0.52 |  |
|  |  |  |  | Italian DMD Boys-early ambulatory | 31 | 0.9 |  |
|  |  |  |  | Italian DMD Boys-early non ambulatory | 24 | 0.24 |  |
|  |  |  |  | Italian DMD Boys-late ambulatory | 35 | 0.72 |  |
|  |  |  |  | Italian DMD Boys-late non ambulatory | 32 | 0.14 |  |
|  | UK |  |  | UK DMD Boys | 191 | 0.43 |  |
|  |  |  |  | UK DMD Boys-early ambulatory | 46 | 0.65 |  |
|  |  |  |  | UK DMD Boys-early non ambulatory | 34 | 0.25 |  |
|  |  |  |  | UK DMD Boys-late ambulatory | 62 | 0.59 |  |
|  |  |  |  | UK DMD Boys-late non ambulatory | 49 | 0.14 |  |
|  | US |  |  | US DMD Boys | 284 | 0.45 |  |
|  |  |  |  | US DMD Boys-early ambulatory | 48 | 0.72 |  |
|  |  |  |  | US DMD Boys-early non ambulatory | 49 | 0.21 |  |
|  |  |  |  | US DMD Boys-late ambulatory | 110 | 0.63 |  |
|  |  |  |  | US DMD Boys-late non ambulatory | 77 | 0.18 |  |
| Pentek, 2014[^49^](#_ENREF_49) | Hungary | NR | EQ-5DL | All DMD | 57 | 0.31 | 0.198 |

Table A15: Main features of cost of illness studies

| **First author & publication year** | **Data collection years** | **Country** | **Name of subgroup** | **Cost Year** | **Currency** | **Direct Health Care, Indirect Costs and Social Care Costs** | **Out of Pocket Costs** | **No. of patients** | **Median age (yrs)** | **Lower IQR (yrs)** | **Upper IQR (yrs)** |
| --- | --- | --- | --- | --- | --- | --- | --- | --- | --- | --- | --- |
| Landfeldt(a), 2014[^58^](#_ENREF_58) | 2012 to 2013 | Germany | DMD Age 9 to 17 | 2012 | US dollar ($) | Yes | Yes | 173 | 13 | 9 | 17 |
|  |  | Italy | DMD Age 8 to 17 | 2012 | US dollar ($) | Yes | Yes | 122 | 12 | 8 | 17 |
|  |  | UK | DMD Age 8 to 17 | 2012 | US dollar ($) | Yes | Yes | 191 | 12 | 8 | 17 |
|  |  | US | DMD Age 9 to 17 | 2012 | US dollar ($) | Yes | Yes | 284 | 12 | 9 | 17 |
| Larkindale, 2014[^32^](#_ENREF_32) | 2008 to 2010 | US | DMD Age 0 to 64* | 2010 | US dollar ($) | Yes | No | 2165 | NR | NR | NR |
| Schreiber-Katz, 2014[^23^](#_ENREF_23) | 2013 | Germany | DMD Age 1 to 42 | 2013 | Euro (€) | Yes | No | 248 | 11 | NR | NR |
|  |  |  | Stage 1 DMD Age 1 to 14 | 2013 | Euro (€) | Yes | No | 49 | 4 | NR | NR |
|  |  |  | Stage 2 DMD Age 3 to 14 | 2013 | Euro (€) | Yes | No | 70 | 7.5 | NR | NR |
|  |  |  | Stage 3 DMD Age 10 to 23 | 2013 | Euro (€) | Yes | No | 11 | 13 | NR | NR |
|  |  |  | Stage 4 DMD Age 1 to 31 | 2013 | Euro (€) | Yes | No | 92 | 16 | NR | NR |
|  |  |  | Stage 5 DMD Age 11 to 40 | 2013 | Euro (€) | Yes | No | 26 | 22.5 | NR | NR |

* A cost of illness survey on a smaller population with no age limits was used for family and society cost estimates
