# Supplementary material for: The burden, epidemiology, costs and treatment for Duchenne muscular dystrophy: an evidence review
Source: Orphanet J Rare Dis. 2017 Apr 26;12:79. doi: 10.1186/s13023-017-0631-3 (PMC5405509; doi:10.1186/s13023-017-0631-3)
Supplement: Supplementary file 5 — Summary of included studies by research question [64–73]. (DOC 462 kb) [file 13023_2017_631_MOESM5_ESM.doc]

Additional file 1: Summary of included studies by research question

| **First author & publication year** | **Prevalence/ Mortality** | **Guidelines** | **Diagnostics** | **Ambulatory** | **Respiratory** | **Cardiac** | **Intellection** | **Utility** | **Cost of illness** | **Treatment** |
| --- | --- | --- | --- | --- | --- | --- | --- | --- | --- | --- |
| Ashwath, 2014[^37^](#_ENREF_37) | - | - | Yes | - | - | Yes | - | - | - | - |
| Baiardini, 2011[^56^](#_ENREF_56) | - | - | - | - | - | - | - | Yes | - | - |
| Bello(a), 2015[^25^](#_ENREF_25) | - | - | Yes | Yes | - | - | - | - | - | Yes |
| Bendixen, 2012[^53^](#_ENREF_53) | - | - | - | - | - | - | - | Yes | - | - |
| Bendixen, 2014[^51^](#_ENREF_51) | - | - | - | - | - | - | - | Yes | - | - |
| Birnkrant, 2010[^59^](#_ENREF_59) | - | Yes | - | - | - | - | - | - | - | - |
| Bladen, 2015[^65^](#_ENREF_65) | - | - | Yes | - | - | - | - | - | - | - |
| Bushby(d), 2010[^6^](#_ENREF_6) | - | Yes | - | - | - | - | - | - | - | - |
| Connolly, 2013[^41^](#_ENREF_41) | - | - | Yes | - | - | - | Yes | - | - | - |
| Davidson, 2014[^5^](#_ENREF_5) | - | - | - | Yes | Yes | - | - | - | - | - |
| de Moura, 2015[^29^](#_ENREF_29) | - | - | - | Yes | - | - | - | Yes | - | - |
| Fox, 2015[^48^](#_ENREF_48) | - | - | - | - | - | - | - | - | - | Yes |
| Henricson, 2012[^66^](#_ENREF_66) | Yes | - | Yes | FU | - | - | - | - | - | Yes |
| Henricson, 2013[^21^](#_ENREF_21) | - | - | - | FU | - | - | - | Yes | - | - |
| Houwen-van Opstal, 2014[^57^](#_ENREF_57) | - | - | - | - | - | - | - | Yes | - | - |
| Janssen, 2014[^36^](#_ENREF_36) | - | - | - | Yes | - | - | - | - | - | Yes |
| Kempen, 2014[^67^](#_ENREF_67) | - | - | - | Yes | - | - | - | - | - | - |
| Khirani, 2014[^35^](#_ENREF_35) | - | - | Yes | - | FU | - | - | - | - | Yes |
| Kieny, 2013[^17^](#_ENREF_17) | Yes | - | Yes | - | FU | - | - | - | - | Yes |
| Kinnett, 2015[^11^](#_ENREF_11) | - | Yes | - | - | - | - | - | - | - | - |
| Landfeldt(a), 2014[^58^](#_ENREF_58) | - | - | - | - | - | - | - | Yes | Yes | - |
| Larkindale, 2014[^32^](#_ENREF_32) | - | - | - | Yes | Yes | - | - | - | Yes | - |
| Lerario, 2012[^68^](#_ENREF_68) | - | - | Yes | Yes | - | - | - | - | - | - |
| Lim, 2014[^50^](#_ENREF_50) | - | - | - | - | - | - | - | Yes | - | - |
| Lorusso, 2013[^31^](#_ENREF_31) | - | - | Yes | Yes | - | - | Yes | - | - | - |
| Magri(b), 2011[^30^](#_ENREF_30) | - | - | Yes | Yes | - | - | - | - | - | - |
| Magri(a), 2011[^4^](#_ENREF_4) | - | - | Yes | Yes | - | - | - | - | - | Yes |
| Mah, 2011[^15^](#_ENREF_15) | Yes | - | - | - | - | - | - | - | - | - |
| Mah, 2012[^44^](#_ENREF_44) | - | - | - | FU | - | - | - | - | - | - |
| Martigne, 2011[^28^](#_ENREF_28) | Yes | - | Yes | Yes | FU | - | - | - | - | - |
| Mayer, 2015[^27^](#_ENREF_27) | - | - | Yes | Yes | Yes | - | - | - | - | Yes |
| McDonald(d), 2013[^22^](#_ENREF_22) | - | - | Yes | FU | - | - | - | - | - | - |
| McDonald(e), 2013[^69^](#_ENREF_69) | Yes | - | Yes | - | - | - | - | - | - | Yes |
| Mendell, 2012[^2^](#_ENREF_2) | Yes | - | Yes | - | - | - | - | - | - | - |
| Moat, 2013[^3^](#_ENREF_3) | Yes | - | - | - | - | - | - | - | - | - |
| Nakamura, 2013[^26^](#_ENREF_26) | - | - | Yes | Yes | Yes | - | - | - | - | Yes |
| Norwood FL, 2009[^12^](#_ENREF_12) | Yes | - | Yes | - | - | - | - | - | - | - |
| Pane(a), 2014[^34^](#_ENREF_34) | - | - | - | FU | - | - | - | - | - | Yes* |
| Pane(b), 2014[^42^](#_ENREF_42) | - | - | Yes | FU | - | - | - | - | - | - |
| Pangalila(a), 2015[^54^](#_ENREF_54) | - | - | - | - | - | - | - | Yes | - | - |
| Passamano, 2012[^18^](#_ENREF_18) | Yes | - | Yes | - | - | - | - | - | - | - |
| Pentek, 2014[^49^](#_ENREF_49) | - | - | - | - | - | - | - | Yes | - | - |
| Rall, 2012[^19^](#_ENREF_19) | Yes | - | Yes | Yes | Yes | - | - | - | - | - |
| Rasmussen, 2012[^13^](#_ENREF_13) | Yes | - | - | - | - | - | - | - | - | - |
| Ricotti, 2012[^47^](#_ENREF_47) | - | - | - | - | - | - | - | - | - | Yes |
| Roberto, 2011[^70^](#_ENREF_70) | - | - | Yes | - | Yes | Yes | - | - | - | Yes |
| Rodger, 2015[^39^](#_ENREF_39) | - | - | - | Yes | Yes | - | - | - | - | Yes |
| Romitti, 2015[^14^](#_ENREF_14) | Yes | - | - | - | - | - | - | - | - | - |
| Sarrazin, 2014[^71^](#_ENREF_71) | - | - | Yes | - | - | - | - | - | - | Yes |
| Schreiber-Katz, 2014[^23^](#_ENREF_23) | - | - | Yes | - | - | - | - | Yes | Yes | Yes |
| Seferian, 2015[^72^](#_ENREF_72) | - | - | Yes | FU | Yes | Yes | - | - | - | - |
| Simon, 2011[^55^](#_ENREF_55) | - | - | - | - | - | - | - | Yes | - | - |
| Soderpalm, 2012[^45^](#_ENREF_45) | - | - | Yes | FU | - | - | - | - | - | Yes |
| Spurney, 2014[^24^](#_ENREF_24) | - | - | Yes | - | - | Yes | - | - | - | - |
| Thomas, 2012[^38^](#_ENREF_38) | - | - | Yes | - | - | FU | - | - | - | - |
| Uzark, 2012[^52^](#_ENREF_52) | - | - | - | - | - | - | - | Yes | - | - |
| Vry, 2013[^40^](#_ENREF_40) | - | - | - | - | Yes | - | - | - | - | Yes |
| West, 2013[^73^](#_ENREF_73) | - | - | Yes | - | - | - | - | - | - | - |

Note Yes = Contributory evidence, FU = Contributory evidence with follow up data

* Reported in Mazzone, 2011[^74^](#_ENREF_74)
